# Taxonomy of the major rhizomorphic species of the “Melanopus group” within Polyporaceae in Yasuní National Park, Ecuador

**DOI:** 10.1371/journal.pone.0254567

**Published:** 2021-08-04

**Authors:** Cristina E. Toapanta-Alban, María E. Ordoñez, Charles W. Barnes, Robert A. Blanchette

**Affiliations:** 1 Department of Plant Pathology, University of Minnesota, St. Paul, Minnesota, United States of America; 2 QCAM Fungarium, Pontificia Universidad Católica del Ecuador, Quito, Ecuador; 3 Forest Health Protection-Region 5, USDA Forest Service, San Bernardino, California, United States of America; Sichuan University, CHINA

## Abstract

Yasuní National Park in Ecuador is one of the most biodiverse places on earth. The fungi in this tropical rainforest are also diverse but have received little research attention. This research paper focuses on an important group of fungi in the family Polyporaceae and examines the genera *Polyporus*, *Atroporus*, and *Neodictyopus* that form aerial melanized cord-like structures called rhizomorphs. Phylogenetic analyses, macro and micromorphological descriptions of basidiomata and rhizomorphs, as well as cultural characterization were completed to better understand these ecologically important fungi. Here we describe four new species: *Atroporus yasuniensis*, *Atroporus tagaeri*, *Neodictyopus sylvaticus*, and *Polyporus taromenane*, and a new variety *Polyporus leprieurii* var. *yasuniensis*. The information presented in this study adds important new knowledge about the unusual rhizomorph producing fungi found in Yasuní National Park, Ecuador and other tropical rainforests.

## Introduction

Rhizomorphs are fully independent organs formed of parallel hyphal aggregations with different levels of hyphal specialization [1–3]. They are often covered by a black cuticle of melanin for protection against microbial antagonism, UV light and other environmental hazards [2, 4, 5]. Rhizomorphs can transport air, water, and solutes by using a complex network system that responds to gravity, light, carbon dioxide and changes in nutrient availability [2, 3, 5]. The development of rhizomorphs can confer advantages for survival in harsh and highly colonized environments, allowing the rhizomorphic fungus to explore and colonize new substrates, extract and store resources, and promote infection of woody plants [3]. Additionally, they can be a survival mechanism allowing the fungus to survive much longer than spores and basidiomata [6, 7].

These structures have been reported to be produced by fungi across 54 families in the Basidiomycota [2]. However, it is more likely this number is underestimated as these structures are often misidentified as plant roots or not associated with their true basidiome. In temperate forests, underground rhizomorphic fungi have been reported to be saprophytic and parasitic on trees. Some, such as the *Armillaria* species complex, cause significant damage to trees and other woody plants [6, 8, 9]. In contrast, tropical rhizomorphic fungal species, especially those that produce rhizomorphs above ground are poorly understood. A few studies have been completed on the aerial rhizomorphs produced by fungi in the Marasmiaceae family and their symbiotic associations with birds [10–12].

The genus *Polyporus* was first proposed in 1729 by Pier Antonio Micheli. Fries (1820) used this genus name for 130 stipitate, pileate, and resupinate species, widening Micheli’s concept [4, 13]. Early classification was based on macromorphological characteristics such the presence or absence of a stipe and the context of the pileus [4, 13]. *Polyporus tuberaster* (Jacq. Ex Pers.) Fr., the type species of the genus, is described as a fleshy, stipitate basidiome with dimitic hyphal system; hyaline, smooth, cylindrical to sub-ellipsoid basidiospores with no reaction to Melzer’s reagent; terrestrial growing on exposed or buried wood causing white rot in wood and having wide global distribution [4, 13]. Núñez and Ryvarden (1995), divided *Polyporus* into six morphological groups to segregate species that shared marked macromorphological characteristics. These included *Polyporus*, *Favolus*, *Melanopus*, *Polyporellus*, *Admirabilis* and *Dendropolyporus* [4]. *Favolus* P. Beavuv. (1805), *Polyporellus* P. Karst. (1879), and *Dendropolyporus* (Pouzar) Jülich (1982) were previously accepted as independent genera. With the advancement of molecular techniques, *Polyporus* underwent important taxonomic changes and has been identified as a polyphyletic genus [14–16]. Several taxa within the morphological groups have been newly amended, proposed and accepted, as independent genera within the “Melanopus” group. The new genera are *Atroporus* Ryvarden (1973) [17], *Picipes* Zmitr. & Kovalenko (2016) [18], and *Neodictyopus* Palacio, Robledo & Drechsler-Santos (2017) [17]. We believe it is important to continue decrypting the species described within *Polyporus* P. Micheli ex Adans that do not match Micheli’s original description but are part of the original morphological groups, in order to unveil their true genetic relationships.

The species that are being analyzed in this work share morphological features of the “Melanopus group” but have not been studied previously. The presence of rhizomorphs has not been established in prior phylogenetic studies of the “Melanopus group” [14–18] and in taxonomic studies, rhizomorphs are considered to not have taxonomic purposes [4, 15, 16]. Our research focused on the taxonomy of several ecologically important *Polyporus* sensu lato species that are ubiquitous in the forest of one of the most biodiverse places on earth, Yasuní National Park in the Ecuadorian Amazon. Identifying these rhizomorphic fungi are a first step to better understand their role in the Amazon rainforest of Ecuador and in other ecosystems worldwide, where similar species are found. This paper reports four new species: *Atroporus yasuniensis*, *Atroporus tagaeri*, *Neodictyopus sylvaticus*, and *Polyporus taromenane*, and a new variety *Polyporus leprieurii* var. *yasuniensis*.

## Material and methods

A random stratified sampling method was used to survey and collect fungal samples (basidiomata and rhizomorphs) in the 50 ha of the Yasuní Forest Dynamic Plot (YFDP), in the Yasuní National Park, Ecuador (0° 41’ 0.5” S 76° 23’ 58.9” W) (https://forestgeo.si.edu/sites/neotropics/yasuni). Ten quadrants of 100x100 m and six transects of 100x20 m were selected randomly over the 50 ha to cover a significant proportion of the plot that includes all topographical habitats: ridges, slopes and valleys (temporary flooded and terra firme forest) [19]. Six surveys were carried out (December 2016, July 2017, December 2018, July 2019, October 2019 and December 2019). Collection sites were marked and monitored during each visit to record the production of basidiomata and presence/absence of rhizomorphs associated with these fungi within the sampling years. Research was carried out under a QCAM Fungarium research permit of Pontificia Universidad Católica del Ecuador in Quito, where the samples were accessioned. Two additional samples, EC-97, a culture and herbarium sample from Los Salideros, Cuyabeno National Park-Ecuador and BZ-958, a herbarium sample from Blue-Hole National Park-Belize, were obtained from the USDA Forest Products Laboratory, Center for Forest Mycology Research, Wisconsin, USA.

### Morphology

Macro-morphological descriptions and ecological observations were made in the field. Substrates were identified as fine woody debris (FWD) when wood diameters were 0.5–9 cm and coarse woody debris (CWD) when wood diameters were ≥ 10 cm. When growing on a living tree, the trees were identified from the Forest Dynamic Laboratory database, Pontificia Universidad Católica del Ecuador. Color terms used followed the “Mycological Colour Chart” and “Munsell soil color charts” [20, 21]. Micro-morphological characters were observed under a compound light microscope (Nikon E600®) using standard methods for the describing polypores [22]. Freehand cross sections of dried samples mounted with Melzer’s reagent, and/or, 3% NaOH, 5 and 10% KOH, sterile water, 1% Congo red, 1% phloxine, and 75% glycerol were prepared in order to observe spores. For hyphal systems and other individual structures, cross sections in 3% NaOH were incubated in a 60° C water bath for four hours, then vigorously shaken for one min at 20000 speed in the MagNA Lyser Roche®, dissected under the stereomicroscope (Nikon SM2800®) and mounted on water and/or the reagents listed above. Basidiospores and other structures were measured using a light microscope at 100x magnification with deionized water and/or reagents (n = 40 to 100 depending on the number of structures available within each sample). For basidiospores, shape terminology was that of the Stalpers database (https://russulales.mycobank.org/). Abbreviations are as follow: IKI+ = dextrinoid, IKI- = inamyloid and indextrinoid, CB+/- = cyanophilous/acyanophilous, ave = arithmetic mean, Q = the ratio of length/width of basidiospores.

### Fungal isolations and cultural characterization

Using aseptic techniques, small pieces of wood substrates (~5 mm), from areas where rhizomorphs and basidiomata were attached to wood, were placed in 0.5% NaOCl solution for one minute, rinsed with running sterile water for ten seconds, and excess solution was removed with clean paper towels. The small pieces of wood were then placed in petri dishes containing malt agar with additives to prevent the growth of bacteria and contaminating Ascomycota. A semi-selective media (BSA) was used containing 15 g malt extract, 15 grams of agar, 2 g of yeast extract, 0.05 g benomyl, 0.5 g streptomycin and 1 L deionized water [23]. Isolates were monitored daily for growth and when mycelium extended 10mm, they were transferred to new plates. Fungal isolates were used for DNA extractions and to determine cultural characteristics.

For cultural characterization, two culturing media were used including malt extract agar (MEA) 15 g malt extract, 15 g of agar, 1 L deionized water and malt yeast agar (MYA) 15 g malt extract, 15 grams of agar, 2 g of yeast extract and 1 L deionized water [23]. Isolates were incubated at 24–26 C°. Six plates were used for each type of media. After five days, growth (mm) was determined using a digital caliper. Hyphal density, margins of the colony, color, and hyphal melanization were noted. Morphological descriptions and terms followed Stalpers (1978) [24].

### DNA extraction, PCR amplification and sequencing

DNA extractions were made from pure cultures grown on MYA and/or from rhizomorphs, stipe, and/or pileus samples preserved in 95% ethanol following the CTAB extraction protocol Of Blanchette et al. 2016 [23]. When DNA extractions were from pure cultures, ¼ of fungal tissue on the petri (10 mm diameter) dish was scraped. When isolations were from other tissues, a 5 mm piece of tissue was cut into smaller pieces. Fungal tissue was suspended in 500 μl of cetyltrimethylammonium bromide (CTAB) lysis buffer with glass beads and vortexed for 1 minute for hyphal tissue and 5 minutes for pileus/stipe/rhizomorphs, samples were centrifuged for 30 seconds to aggregate fungal material. Supernatant was transferred to a new microcentrifuge tube and placed in a hot water bath at 65° C for 20 minutes. Subsequent, 500 μl of chloroform/phenol/isoamyl alcohol (25:24:1) was added to the tubes, shaken vigorously and centrifuged for 5 min at 15,000 rcf. The supernatant was then transferred to a clean microcentrifuge tube and isopropanol (stored at -20° C) was added (2/3 the amount of supernatant), tubes were inverted a few times and incubated at room temperature for 5 min. Tubes were then centrifuged for 7 min at 20,000 rcf, and supernatant was carefully removed. To the remaining DNA pellet, 500 μl of 70% ethanol (stored at -20° C) were added and centrifuged for 3 min at 20,000 rcf to wash the pellet. Ethanol was carefully removed, and tubes were left in a sterilized bio-safety cabinet to air dry. DNA was rehydrated with 100 μl of sterile water.

The partial regions of the nuclear ribosomal small subunit (SSU), nuclear ribosomal internal transcribed spacer (ITS), nuclear ribosomal large subunit (LSU), elongation factor 1-alpha (EF1-α) and the RNA polymerase II largest subunit (RPB1) were amplified by Polymerase Chain Reaction (PCR). The primers used for each region were: PNS1 with NS41 for SSU, ITS1F with ITS4 or ITS4B for ITS, LROR with LR7 or LR5 for LSU, EF1-983F with EF1-1567R for EF1-α and RPB1-Af with rpb1-Cr for RPB1 [25]. The PCR final volume was 25.5 μl, each tube containing 1 μl of DNA template, 1 μl of each primer (10 μM for RPB1 and 5 μM for the other regions), 0.5 μl of bovine serum albumin (BSA), 12.5 μl of GoTaq® Green Master Mix and 9.5 μl of sterile water in a thermocycler. PCR program for ITS and SSU were: 94° C for 5 min, 35 cycles of 94° C for 1 min, and 72° C for 1 minute, followed by a final extension step of 72° C for 5 min. For LSU: 95° C for 3 min, 94° C for 1min, 50° C for 45 sec, 74° C for 2 min ramping up 0.3° C/sec, 94° C for 1 min for 29 cycles and a final step of 72° C for 5 min. For EF1-α and PBB1 the same program was followed as: 94° C for 2 min, 94° C for 40 sec, 60° C for 40 sec decreasing 1° C per cycle, 72° C for 2 min,94° C for 40 sec for 8 cycles, 94° C for 45 sec, 53° C for 1:30 min, 72° C for 2 min, 94° C for 45 sec for 36 cycles and a final step of 72° C for 10 min.

Sequencing was carried out with Big Dye V3.1 and run on ABI 3730xl instruments (Functional Biosciences, Madison, WI, USA). Consensus sequences were assembled using Geneious 7.0 [26]. For samples with multiple DNA origins (pure culture, basidiomata and/or rhizomorphs), individual consensus sequences were created and then aligned to verify the DNA came from the same sample. When alignments had more than 99% pairwise identity, a single consensus sequence was created with all multiple DNA sequences to represent a specific sample. All final consensus sequences were compared through BLAST searches to GenBank sequences, whose identification has been supported with taxonomic publications to prevent sequences matching those wrongly identified. All sequences generated in this study were submitted to GenBank (**Table 1**).

**Table 1:** Species list of *Polyporus* and related genera used in this study and GenBank entries.

No.	Species	Specimen No.	Country	GenBank accession No.				
				ITS	nLSU	EF1-α	RPB1	nSSU
1	*Atroporus diabolicus* BRZ	DS1266	Brazil	KY631768	KY631757	-	-	-
2	*A*. *rufoatratus* BRZ	MP153	Brazil	KY631771	KY631760	-	-	-
3	*A*. *yasuniensis*	CTR-2-46^a^	Ecuador	MT950131	MT950161	MW287083	MW287110	MT950207
4	*A*. *tagaeri*	CTR-2-55 ^a^	Ecuador	MT950129	MT950159	MW287084	-	MT950205
5	*A*. *tagaeri*	CTR-2-11^a^	Ecuador	MT950130	MT950160	MW287109	-	MT950206
6	*Neodictyopus atlanticae BRZ* 1	DS1285	Brazil	KY631762	KY631773	-	-	-
7	*N*. *atlanticae* BRZ 2	DS1286	Brazil	KY631763	KY631774	-	-	-
8	*N*. *gugliottae* BRZ	GAS622	Brazil	KY631761	KY631772	-	-	-
9	*N*. *dictyopus* BRZ 1	GAS60	Brazil	KY631765	KY631776	-	-	-
10	*N*. *dictyopus* BRZ 2	GAS281	Brazil	KY631767	KY631778	-	-	-
11	*N*. *sylvaticus*	CTR-2-60 ^a^	Ecuador	MT950155	MT950183	MW287085	MW287111	MT950229
12	*Neodictyopus* sp.	EC-97 ^a^	Ecuador	MT950132	-	-	-	-
13	*Picipes badius* CHN 1	Cui 10853	China	KU189780	KU189811	KU189929	KU189894	KU189844
14	*P*. *badius* CHN 2	Cui 11136	China	KU189781	KU189812	KU189930	KU189895	KU189845
15	*Pi*. *baishanzuensis* CHN	Dai 1318 (T)	China	KU189762	KU189793	KU189907	KU189882	KU189823
16	*Pi*. *conifericola* CHN	Cui 9950	China	KU189783	KU189814	KU189934	KU189897	KU189848
17	*Pi*. *rhizophilus* CHN	Dai 11599	China	KC572028	KC572067	KU189933	KU189896	KU189847
18	*Pi*. *submelanopus* CHN	Dai 13294	China	KU189770	KU189801	KU189951	KU189886	KU189830
19	*Pi*. *subtropicus* CHN	Li 1928	China	KU189758	KU189790	KU189904	KU189881	KU189820
20	*Pi*. *virgatus* ARG 1	CulTENN11406	Argentina	AF516582	AJ488123	-	-	-
21	*Pi*. *virgatus* ARG 2	CulTENN11219	Argentina	AF516581	AJ488122	-	-	-
22	*Polyporus dictyopus* BLZ	TENN59385	Belize	AF516561	AJ487945	-	-	-
23	*P*. *guianensis* ARG	TENN 59093	Argentina	AF516564	AJ487947	-	-	-
24	*P*. *guianensis* VNZ	TENN 58404	Venezuela	AF516566	AJ487948	-	-	-
25	*P*. *guianensis* ECU 1	665	Ecuador	KP133243	-	-	-	-
26	*P*. *guianensis* ECU 2	899	Ecuador	KP133245	-	-	-	-
27	*P*. *guianensis* CRI	CulTENN10064	Costa Rica	AF516565	-	-	-	-
28	*P*. *taromenane*	CTR-1-8 ^a^	Ecuador	MT950126	MT950157	MW287086	MW287112	MT950202
29	*P*. *taromenane*	CTR-2-42 ^a^	Ecuador	MT950127	MT950158	MW287087	MW287113	MT950203
30	*P*. *taromenane*	CTR-3-21 ^a^	Ecuador	MT950128	-	MW287088	MW287114	MT950204
31	*P*. *hapalopus* CHN	Yuan 5809	China	KC297219	KC297220	KU189918	-	KU189833
32	*P*. *hemicapnodes* 1	Dai 13404	China	KX851626	KX851680	KX851783	-	-
33	*P*. *hemicapnodes* 2	Cui 11259	China	KX851625	KX851679	KX851782	-	KX851733
34	*P*. *hemicapnodes* 3	Dai 13403	China	KX851627	KX851681	KX851784	-	KX851734
35	*P*. *leprieurii* CRI	TENN58597	Costa Rica	AF516567	AJ487949	-	-	-
36	*P*. *leprieurii* var. *yasuniensis*	CTR-2-12 ^a^	Ecuador	MT950143	MT950172	MW287089	MW287115	MT950218
37	*P*. *leprieurii* var. *yasuniensis*	CTR-2-23 ^a^	Ecuador	MT950151	MT950180	MW287090	MW287116	MT950226
38	*P*. *leprieurii* var. *yasuniensis*	CTR-2-67 ^a^	Ecuador	MT950135	MT950164	MW287091	MW287117	MT950210
39	*P*. *leprieurii* var. *yasuniensis*	CTR-2-14 ^a^	Ecuador	MT950133	MT950162	MW287092	MW287118	MT950208
40	*P*. *leprieurii* var. *yasuniensis*	CTR-2-27 ^a^	Ecuador	MT950134	MT950163	MW287093	MW287119	MT950209
41	*P*. *leprieurii* var. *yasuniensis*	CTR-2-65 ^a^	Ecuador	MT950147	MT950176	MW287094	-	MT950222
42	*P*. *leprieurii* var. *yasuniensis*	CTR-2-57 ^a^	Ecuador	MT950141	MT950170	MW287095	MW287120	MT950216
43	*P*. *leprieurii* var. *yasuniensis*	CTR-2-18 ^a^	Ecuador	MT950150	MT950179	MW287096	-	MT950225
44	*P*. *leprieurii* var. *yasuniensis*	CTR-2-35 ^a^	Ecuador	MT950145	MT950174	MW287097	MW287121	MT950220
45	*P*. *leprieurii* var. *yasuniensis*	CTR-3-10 ^a^	Ecuador	MT950152	MT950181	MW287098	-	MT950227
46	*P*. *leprieurii* var. *yasuniensis*	CTR-2-33 ^a^	Ecuador	MT950144	MT950173	MW287099	MW287122	MT950219
47	*P*. *leprieurii* var. *yasuniensis*	CTR-3-18 ^a^	Ecuador	MT950142	MT950171	MW287100	MW287123	MT950217
48	*P*. *leprieurii* var. *yasuniensis*	CTR-3-23 ^a^	Ecuador	MT950153	MT950182	-	-	MT950228
49	*P*. *leprieurii* var. *yasuniensis*	CT-16F ^a^	Ecuador	MT950148	MT950177	MW287101	MW287124	MT950223
50	*P*. *leprieurii* var. *yasuniensis*	CTR-1-7.2^a^	Ecuador	MT950149	MT950178	MW287102	MW287125	MT950224
51	*P*. *leprieurii* var. *yasuniensis*	CTR-2-61 ^a^	Ecuador	MT950146	MT950175	MW287103	MW287126	MT950221
52	*P*. *leprieurii* var. *yasuniensis*	CTR-2-51 ^a^	Ecuador	MT950136	MT950165	MW287104	MW287127	MT950211
53	*P*. *leprieurii* var. *yasuniensis*	CTR-2-49 ^a^	Ecuador	MT950139	MT950168	MW287105	MW287128	MT950214
54	*P*. *leprieurii* var. *yasuniensis*	CTR-2-31 ^a^	Ecuador	MT950138	MT950167	MW287106	MW287129	MT950213
55	*P*. *leprieurii* var. *yasuniensis*	CTR-2-39 ^a^	Ecuador	MT950140	MT950169	MW287107	MW287130	MT950215
56	*P*. *leprieurii* var. *yasuniensis*	CTR-1-6.1^a^	Ecuador	MT950137	MT950166	MW287108	MW287131	MT950212
57	*P*. *leprieurii*	BZ-958 ^a^	Belize	MT950154	-	-	-	-
58	*P*. *squamosus* CHN	Cui 10595	China	KU189778	KU189809	KU189925	KU189892	KU189840
59	*P*. *squamosus* USA	AFTOL-ID	USA	DQ267123	AY629320	-	-	-
60	*P*. *subvarius*	Yu 2 (T)	China	AB587632	AB587621	KU189924	-	KU189839
61	*P*. *tuberaster* CHN 1	Dai 12462	China	KU507580	KU507582	KU507590	-	KU507586
62	*P*. *tuberaster* CHN 2	Dai 11271	China	KU189769	KU189800	KU189914	-	KU189829
63	*P*. *umbellatus* CHN	Pen 13513	China	KU189772	KU189803	KU189917	KU189887	KU189832
64	*P*. *varius* CHN 1	Cui 12249	China	KU507581	KU507583	KU507591	KU507589	KU507587
65	*P*. *varius* CHN 2	Dai 12249	China	KU189777	KU189808	KU189923	KU189891	KU189838
66	*Trametes conchifer* USA	FP 106793sp	USA	JN164924	JN164797	JN164887	JN164823	-

^a^ Sequences generated in this study.

### Phylogenetic analyses

All 135 newly generated sequences were deposited in GenBank, 28 SSU, 30 ITS, 28 LSU, 27 EF1, and 22 RPB1 (**Table 1**). However, not all sequences were used in the phylogenetic analysis but are available in GenBank for future taxonomic studies. SSU sequences were not included in the analyses as a multiple alignment of the region had 93% identical sites among all taxa, resulting in individual phylogenetic trees without clear delimitation of taxa. Ninety-six sequences from other studies available in GenBank were used in the analyses (**Table 1**).

Two datasets were constructed, the first based on two molecular markers (ITS and LSU), and the second on four molecular markers (ITS, LSU, RPB1, and EF1-α). Datasets included sequences generated in this study and reference sequences of *Polyporus* and allied genera downloaded from GenBank. Individual datasets for each region were aligned using Clustal Omega [27] sequence aligning tool and then manually examined and adjusted in Geneious 7.0 [25]. Due to the high variation of sequence length in the LSU alignment, sequences were trimmed to fit the length of most sequences, for shorter sequences, ends were filled with Ns. Alignments for the other regions were trimmed for an even length for all sequences. Annotations were made to identify polymorphisms (Indels and SNPs) on individual alignments of each molecular marker to identify variants frequency. The best-fit evolutionary model for each partition was calculated on JModelTest [28, 29] following the Bayesian Information Criterion (BCI). The Partition Homogeneity Test (PHT) was run on PAUP to test congruence [30]. Maximum-likelihood trees were generated on MEGA 10.05 tool [31] using the General Time Reversible model to choose the tree with the highest log likelihood with 1000 replicates and Gamma Distributed with invariant sites (G+I) for rates among sites. Bayesian Inference was calculated on Geneious 7.0 [26] using the General Time Reversible model, Gamma distribution, and subsampling frequency at 200 trees on a 1,100,000-chain length. Bayesian and Maximum-likelihood trees were matched to present results.

### Nomenclature acts

The electronic version of this article in Portable Document Format (PDF) in a work with an ISSS or ISBN will represent a published work according to the International Code of Nomenclature for algae, fungi, and plants, and hence the new name contained in the electronic publication of a PLOS article are effectively published under that code from electronic edition alone, there is no longer any need to provide printed copies.

In addition, new names contained in this work have been submitted to MycoBank from which they will be made available to the Global Names Index. The unique MycoBank number can be resolved, and the associated information viewed through any standard web browser by appending the MycoBank number contained in this publication to the prefix http://www.mycobank.org/MB/. The online version of this work is archived and available from the following digital repositories: PubMed Central and LOCKSS.

## Results

Observations of collections from Yasuní National Park indicated that several *Polyporus*-like species were associated with the aerial formation of rhizomorphs. These fungal structures are common on the forest floor and surveys found 109 collection sites with *Polyporus*-like basidiomata samples. Rhizomorphs were associated with these *Polyporus* species in 100 of the sample sites. Differences in morphological characteristics were found among samples, suggesting that several previously undescribed and/or misidentified species were present in this ecosystem. Phylogenetic studies were needed to elucidate the genetic relationships among the sampled taxa and their taxonomy. We report here the phylogenetic analyses and morphological descriptions for the new taxa.

### Phylogenetic analysis

Eighty-eight of the 135 newly generated sequences were used in this study (25 ITS, 21 LSU, 22 EF1-α, and 20 RPB1). Another 96 related sequences (including 34 ITS, 32 LSU, 18 EF1-α, and 12 RPB1) used in the phylogenetic analyses were downloaded from GenBank and are listed in **Table 1**. Datasets were concatenated as follow: ITS-LSU resulted in an alignment with 1468 total characters (including 618 ITS + 850 LSU nucleotides) and ITS-LSU-RPB1-EF1- α with 3267 total characters (including 618 ITS + 850 LSU + 1232 RPB1 + 567 EF1-α nucleotides). The partition homogeneity test indicated that sequences in both datasets were not congruent (P-value = 0.01). In the first dataset, 756 characters were constant (proportion = 0.5), 120 variable characters were parsimony-uninformative, and 592 characters were parsimony-informative. For the second dataset, 1635 characters were constant (proportion = 0.5), 344 variable characters were parsimony-uninformative, and 1288 characters were parsimony-informative. In the Bayesian inference trees, for both datasets, total chain length was set up as 1,100,000, subsample frequencies at 200, and total samples analyzed were 4951. While in the Maximum-likelihood analysis, GTR+I+G model was used, and partitions were established considering individual markets. A discrete Gamma distribution was used to model evolutionary rate differences among sites (5 categories (+G, parameter = 0.5373)). The bootstrap consensus tree inferred from 1000 replicates where branches corresponding to partitions reproduced in less than 50% bootstrap replicates were collapsed. Initial trees for the heuristic search were obtained automatically by applying Neighbor-Join and BioNJ algorithms to a matrix of pairwise distances estimated using the Maximum Composite Likelihood (MCL) approach, and then selecting the topology with superior log likelihood value [32]. One hundred twenty-one variants were found in the first dataset and four hundred seven variants in the second dataset. SNPs were the most common variants among datasets, being 74% and 85% respectively of the total variants found in each dataset (**Table 2**).

**Table 2:** Variants’ analysis summary for individual alignments, ITS-LSU, and ITS-LSU-RPB1-EF1-α datasets.

Alignment	# Seqs.	Sequence Length	# Variants	% Indel	% SNPs	%GC	% Identical Sites
ITS	58	618	89	22	78	46.5	21.5
LSU	55	850	32	28	72	50.9	42.2
EF1-α	40	567	97	5	95	52.5	43
RPB1	32	1232	189	14	86	53.4	47.7
2 Genes^**a**^	59	1468	121	26	74	48.9	34.3
4 Genes^**b**^	59	3267	407	15	85	50.8	11.5

^**a**^ ITS+LSU

^**b**^ ITS+LSU+EF1-α+RPB1

**Fig 1:**
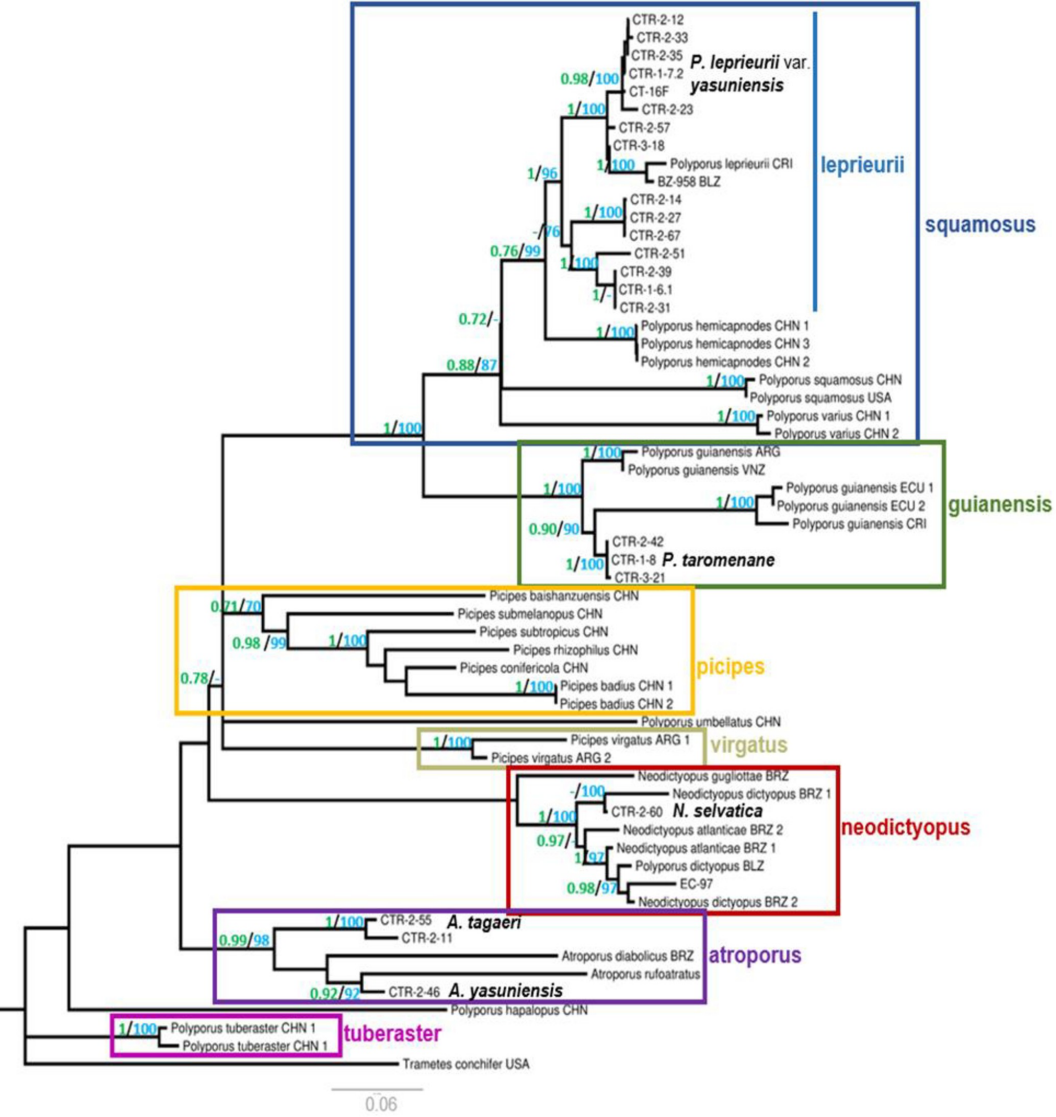
Phylogeny of *Polyporus* and allied genera inferred from ITS+LSU+EF1-α+RPB1 data. Topology is from Maximum-Likelihood analysis with bootstrap support values of nodes on light blue (≥70) and posterior probability values (≥70) of nodes on light green. Bold species are the newly described taxa in this study. ARG = Argentina, BRZ = Brazil, BLZ = Belize, CHN = China, CRI = Costa Rica, ECU = Ecuador, VNZ = Venezuela, and USA = United States.

In both datasets, seven clades (“squamosus”, “guianensis”, “picipes”, “virgatus”, “neodictyopus”, “atroporus”, and “tuberaster”) consistently clustered with bootstraps and posterior probabilities higher than 70% support on nodes. Such clades were named after the genus and/or oldest recognized taxa for an easier identification in the phylogeny (Fig 1).

Samples analyzed in this study nested within the “squamosus”, “guianensis”, “neodictyopus”, and “atroporus” clades, all formed by neotropical taxa. The “Squamosus” and “guianensis” clades contain taxa currently accepted as *Polyporus*, while “neodictyopus” and “atroporus" contain former members of *Polyporus*.

Samples analyzed in this study nested within the “squamosus”, “guianensis”, “neodictyopus”, and “atroporus” clades. Only the “squamosus” and “guianensis” clades contain taxa currently accepted as *Polyporus*, while “neodictyopus” and “atroporus" contain former members of *Polyporus*.

The “squamosus” clade named after *Polyporus squamosus* (Huds.) Fr. was formed by sequences of *P*. *squamosus*, *P*. *varius*, *P*. *hemicapnodes*, *P*. *leprieurii*, and *P*. *leprieurii* var. *yasuniensis* variety novo. The last two formed an internal clade named “leprieurii” after *Polyporus leprieurii* Mont. This clade included samples from Ecuador (Yasuní), Costa Rica, and Belize. We have accepted as the “true” *P*. *leprieurii* the sequences from Costa Rica and Belize for the closer proximity to the type location, the French Guiana, as no sequence is available for the type collection. In this phylogeny, fifteen sequences from Yasuní were closely related to the “true” *P*. *leprieurii*. However, only six samples were designated to the newly proposed *P*. *leprieurii* var. *yasuniensis*, while the remaining sample, forming two well supported related clades within the “leprieurii” clade, need further taxonomical designation. *P*. *hemicapnodes*, an accepted synonym of *P*. *leprieurii* from China is closely related to the “leprieurii” clade, but forms its own well-defined clade (Fig 1).

The “guianensis” clade, named after *P*. *guianensis* Mont., was formed by sequences of *P*. *guianensis* from Argentina and Venezuela; three samples of *P*. *taromenane* species novo from Ecuador; and two samples from Ecuador plus one from Costa Rica previously misidentified as *P*. *guianensis*.

The taxa designated as *Picipes* clustered in two clades: “picipes” named after *Picipes* I.V. Zmitrovich & Kovalenko for having most sequences in this phylogeny, all from China, and “virgatus”, named after *Picipes virgatus* (Berkeley & M.A. Curtis) J.L. Zhou & B.K. Cui which had only two sequences, both from Argentina.

The “neodictyopus” clade, named after the *Neodictyopus* Palacio, Robledo, Reck & Drechsler-Santos genus was formed by taxa from Brazil, Ecuador and Belize. *N*. *sylvaticus* species novo was closely related to one of the sequences of *N*. *dictyopus* from Brazil. The sequence of the herbarium sample, EC-97, was closely related to different sequences of *N*. *dictyopus* from Brazil and *P*. *dictyopus* from Belize.

The “atroporus” clade, named after the *Atroporus* Ryvarden amend. Palacio, Robledo, Reck & Drechsler-Santos genus, included taxa from Brazil and Ecuador. *A*. *tagaeri* and *A*. *yasuniensis* species novo from Ecuador and *A*. *diabolicus* and *A*. *rufoatratus* from Brazil.

Finally, the “tuberaster” clade, named after *Polyporus tuberaster* (Jacquin ex Persoon) Fries consisted of two sequences from China. This clade was found to be very distinct from the other clades. The remaining taxa, *P*. *umbelatus* and *P*. *hapalopus*, both from China, did not cluster with any of the listed clades.

### Taxonomy

***Atroporus*** Ryvarden, Norw. J1 Bot. 20:2 (1973), emend. Palacio, Robledo, Reck & Drechsler-Santos.

Type species: *Polyporus diabolicus* Berk.

Type specimen: Brazil, Spruce 195 (K!)

*Atroporus* was proposed by Ryvarden (1973) based on swollen binding hyphae in the context of the type species, but this characteristic was considered too vaguely defined for a genus description [33]. *Atroporus* was later amended by Palacio, Robledo, Reck & Drechsler-Santos (2017), using basidiospore descriptions and Melzer reagent reaction of the skeletal-binding hyphae in the trama [17].

The genus shares macromorphological characteristics with the “Melanopus group”, which includes annual to biannual basidiomata, coriaceous when fresh and tough when dry; centrally to eccentrically stipitate, with a black cuticle on the stipe. Spores medium to large, 6–12 x 2–4 μm [4].

Description: “Basidiomata annual to biannual, centrally to eccentrically stipitate. Pileus circular; pilear surface glabrous, radially striate to finely wrinkled, dark purplish to red blackish; margins sterile, with a black cuticle. Pores circular. Context homogeneous, light brown. Stipe cylindrical, solid, bearing a black cuticle. Hyphal system dimitic with generative and skeletal-binding hyphae. Generative hyphae with clamp connections. Skeletal-binding from context and stipe abundant, arboriform, hyaline, IKI-. Skeletal-binding hyphae in the trama dextrinoid, with a differentiated wide stalk with sharply pointed apex. Basidia clavate, 4-sterigmate. Basidiospores narrowly ellipsoid to subcylindrical, thin-walled, smooth, hyaline, IKI-” [17].

Remarks: Basidiospores narrowly ellipsoid to subcylindrical, thin-walled, smooth, hyaline, IKI-. Hyphal system dimitic with generative and skeletal-binding hyphae. Generative hyphae present in trama with clamp connections; skeletal-binding hyphae in trama dextrinoid with a distinctive wide stalk with sharply pointed apex that aids in the identification of the genus. All species grow on dead wood, typically on dead fallen branches of relatively thin diameter trees [17].

**Fig 2:**
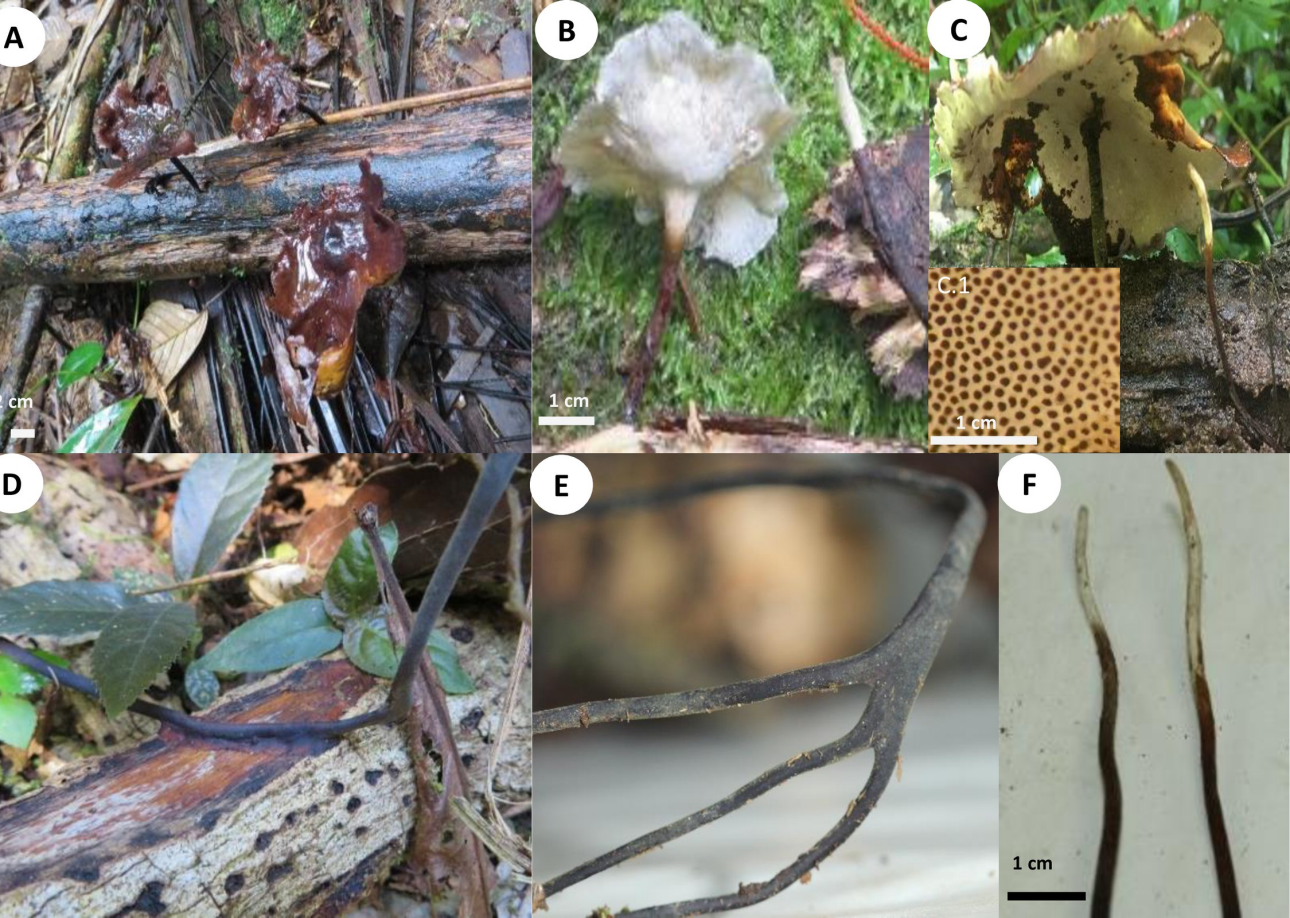
Basidiomata and rhizomorphs of *Atroporus yasuniensis*. **A.** gregarious habit of basidiomata. **B.** young basidiome. **C.** aged basidiome. **C.1.** pore layer. **D.** brown-reddish rhizomorphic mat on decayed wood formed posterior to rhizomorph attachment. **E.** Branching of rhizomorphs. **F.** fresh rhizomorphs with the active growing region white.

**Fig 3:**
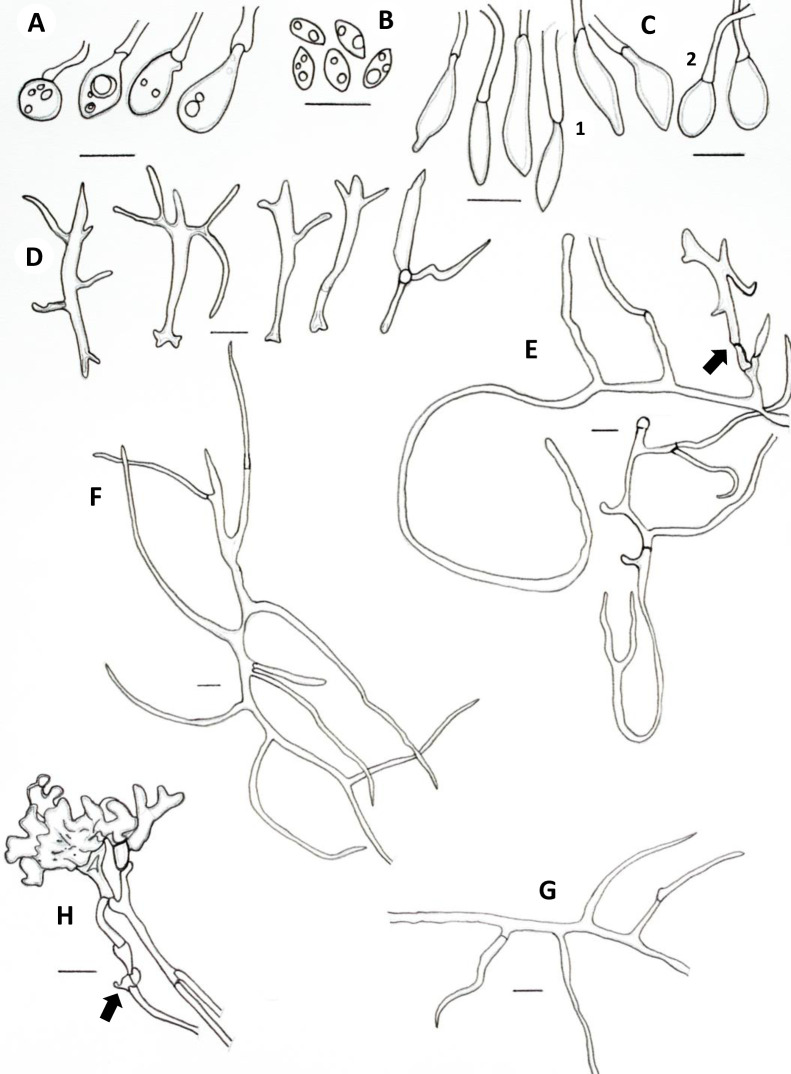
Microscopic features of *A*. *yasuniensis*. **A.** Basidioles. **B.** Basidiospores. **C.** Cystidioles. **C1** of the ventricose type and **C2** oblong-like type. **D.** Thick-walled distinctive stalks of tramal hyphae. **E.** Generative hyphae with a thick-walled distinctive stalk pointed by the arrow. **F.** Skeletal-binding hyphae from context. **G.** Section of skeletal-binding hyphae from stipe. **H.** Generative hyphae from cuticle with melanized cuticle cells (arrow pointing to a clamp connection). Scale bars = 10 μm.

**Fig 4:**
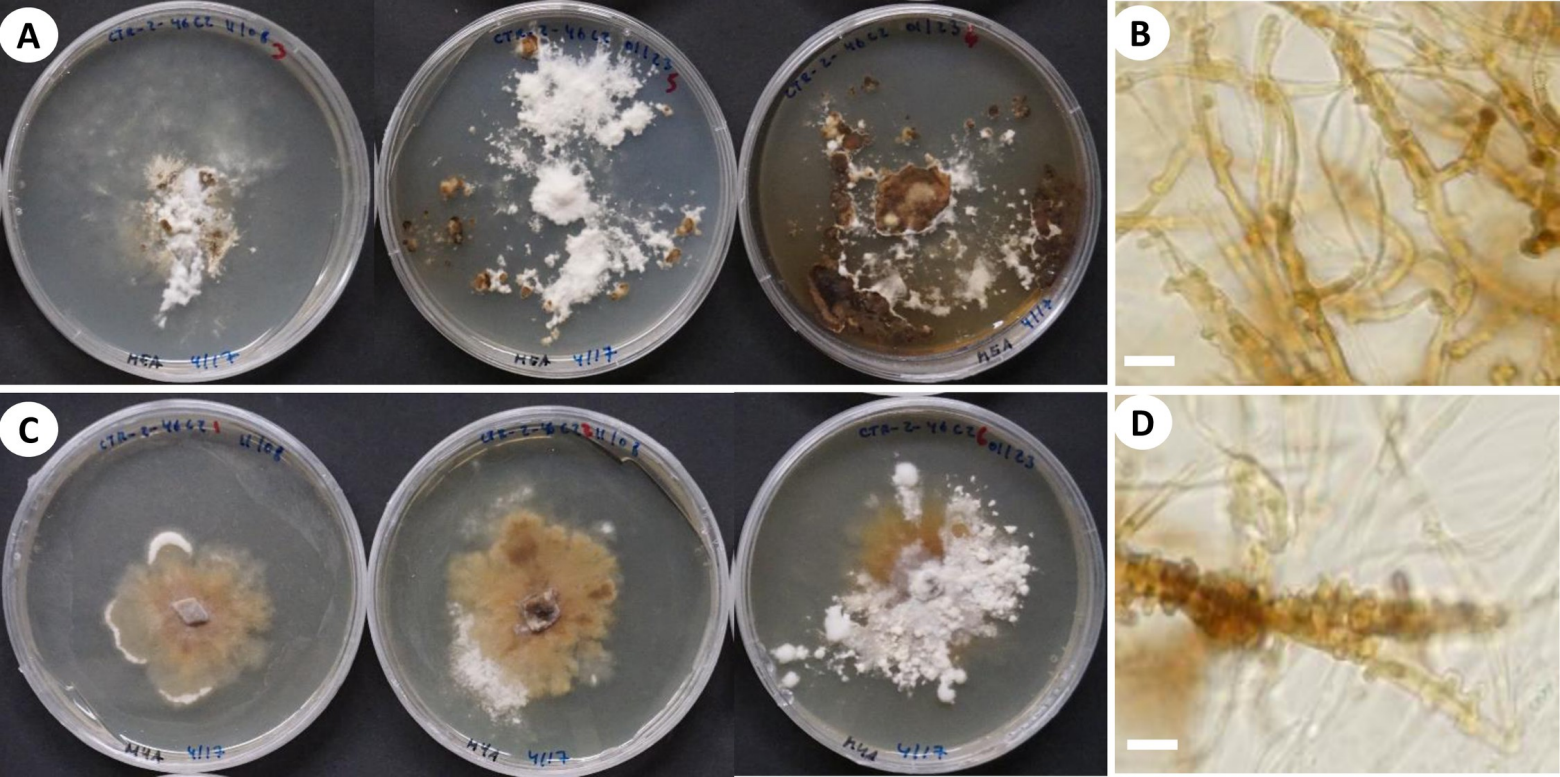
Cultural characteristics and microscopic features of *A*. *yasuniensis*. **A.** macromorphology of colonies on MEA. **B.** Highly branched hyphae with simple septa and clamp connections. **C.** Macromorphology of colonies on MYA. **D.** Generative hyphae with numerous round-like swellings. B and D stained with Melzer’s reagent. Scales bars = 10 μm.

**1. *Atroporus yasuniensis*** Toapanta-Alban, Ordoñez & Blanchette, sp. nov. Figs 2–4.

*Typus*. Ecuador, Orellana Province, Yasuní Forest Dynamic Plot. Collected by C E Toapanta-Alban, 15 Jul 2017, Collection number: CTR-2-46. Found growing on dead wood of angiosperms.

Mycobank no.: MB 837970

Holotype: Fungarium number: QCAM7402 (QCAM)

    Collection number: CTR-2-46, Toapanta-Alban, December 2018

    GenBank no.: (ITS: MT950131), (LSU: MT9501610, (EF1-α: MW287083), (RPB1: MW287110), (SSU: MT950207).

Paratype: Fungarium number: QCAM7402 (QCAM)

    Collection number: CTR-2-46, Toapanta-Alban, July 2019

Etymology: yasuniensis (Yasuní = the paradise/God’s creation (from the Wao Terero language use by the Huaorani/Waorani Indigenous People living in the Amazon Rainforest between the Napo and Curaray Rivers in Ecuador)) + ensis (Latin suffix to denote origin). In reference to the site where samples were collected, Yasuní National Park in Ecuador.

Basidiomata annual, gregarious, centrally stipitate, up to and 6 cm tall. Pilei circular, depressed, infundibuliform with irregular wavy-striate margins that become more pronounced over time; up to 8 cm in diameter and 1.3 mm thick (Fig 2).

Pilear surface smooth, striate, shinny, white to dark-creamy when young; with age the surface remains shinny but becomes glabrous, bay to dark vinaceous (Munsell’s reference: 8. 1R/3.1/6.9; 10. ORP/3.4/4.3). Pilear texture coriaceous, and slightly tough; when dried rigid, slightly shrunken, blood colour to chestnut (Munsell’s reference: 2. 5r/2.3/5.9; 9. 5R/2.3/3.8). Pore surface white when young, creamy when aged, and pale brown when dried. In young specimens, pores are circular, radially arranged; in mature specimens, pores become slightly angular, elongated along the lobular areas near the margin and stipe; 7–10 per mm (ave = 8), 40–80 μm (ave = 59) (Fig 2 C1). Dissepiments entire, 10–38 μm (ave = 21). Tubes concolorous with pore surface, uniform not stratified, up to 0.30 mm; IKI+ reaction with tubes turning bright red. Context homogeneous, light brown, up to 1 mm thick; yellowish-brown in Melzer, weak IKI+ reaction. Stipe cylindrical, robust, solid, glabrous, bearing a lacquered cuticle; dark-reddish when young and black when aged, up to 4.5 mm diameter and up to 4.5 cm long.

Hyphal system dimitic with generative and skeletal-binding hyphae. In the trama, generative hyphae, hyaline, slightly branched with thin-walled sections 1–3 μm, simple septa and clamp connections (Fig 3).

Emerging from the generative hyphae, thick-walled distinctive stalks (up to 4.8 μm thick and 39–52 μm long) with pointing apex and 1–5 branches (1–3 μm) emerging from the middle and apical portion of the stalk; basal clamp connection. Skeletal-binding hyphae found in the trama, context, stipe, and rhizomorphs. Skeletal-binding hyphae in the trama and context of the arboriform type, hyaline, thin and thick-walled 1.5–4 μm, highly branched with simple septa. Skeletal-binding hyphae in the stipe and rhizomorphs are also of the arboriform type, somewhat less branched, hyaline, 1–4 μm thick, with simple septa. In the stipe, generative hyphae thin-walled with clamp connections holding cuticle cells. Cuticle cells brown-yellowish, thick 15–22 μm, 1–4 lobed with rounded ends, forming a palisade layer of groups of three or more cells forming crown-like arrangement. The hymenium is composed of paraphysoids structures (cystidioles, basidioles, and hyphidia) and basidia. Hyphidia, smooth, tubular (5–7μm wide), IKI-. Cystidioles can be of the ventricose type (10–16 x 2.8–8.5 μm) and oblong-like cells (10 x 6 μm). Basidioles abundant, round to clavate (9–16 x 6–8 μm). Basidia, 4-sterigmate, difficult to find free because basidia are encrusted in the hymenophore, only width measurements were recorded (6–8 μm wide). Basidiospores ellipsoid, narrowly ellipsoid to slightly cylindrical, thin-walled, hyaline, smooth, IKI-, 5–7 μm (ave = 6) x 2–4 μm (ave = 3), Q = 1.50–2.5 (ave = 2, n = 40).

Rhizomorphs, circular to slightly flat, solid, robust, coriaceous, thick (up to 5mm diameter), long (up to 3 m), well-branched, bearing a black-reddish lacquered cuticle with white-yellowish tips when fresh (Fig 2). Rhizomorphs are perennial (found growing for over four years in the same area), well distributed along the forest’s floor, emerging from decayed wood (coarse and fine woody debris). The formation of a rhizomorphic mat (brown-reddish tissue that becomes darker over time) facilitates the attachment to new substrates and other plant material on the forest floor. Rhizomorphs undergo anastomosis to form a complex rhizomorphic network. Rhizomorphs use trees and other surfaces to maintain upward growth. They occur on substrates accompanying basidiomata or alone.

Cultural characteristics: Cultures were easily obtained from rhizomorphs and colonized substrates. Initial growth is characterized by white, appressed, silky mycelium that can turn dark brown at different areas of the colony. Growth rate, characteristics of the mycelial mat, presence of aerial mycelium, and color of the colony are variable among colonies growing in the same media.

Growth rate is relatively slow on MEA 10–18 mm (ave = 13.5 mm) compared to MYA 14–31 mm (ave = 19 mm) after one month of growth (Fig 4).

On MEA, the marginal zone is irregular, usually submerged. When mycelium is submerged, pseudosclerotial plates are formed giving the agar a brown coloration and chamois-like appearance. Aerial mycelium is white, floccose, and crustose becoming brown, thick, coriaceous over time. On MYA, margins are regular to irregular, generally appressed, and rarely raised. Mycelium can be submerged and aerial. Aerial mycelium silky, floccose, cottony, white with dark colorations that are not as common as on MEA. Rhizomorphs rarely form on culture media but may be formed after long periods (6–12 months) of growth when nutrients apparently have been utilized (on MYA only).

In cultures, hyphal system is dimitic, with skeletal-binding hyphae in the white-aerial mycelium and generative hyphae in the melanized-crustose sections of mycelium. Skeletal-binding hyphae, highly to slightly branched, thin (1–2.5 μm wide) and thick-walled (2–4.5 μm wide); with simple septa; weakly IKI+ reaction as hyphae turned brown in the reagent; KOH-. Generative hyphae on crustose sections of mycelium are yellowish-brown to brown-reddish, thick-walled (2–8 μm wide) with abundant round-like swellings and clamp connections.

Substrate: Basidiomata and rhizomorphs emerge from decayed fine and coarse woody debris of angiosperms.

Distribution: known from the Amazon rainforest of Ecuador along valleys (temporarily flooded forests).

Specimens examined: Ecuador-Francisco de Orellana Province, Yasuní National Park (0° 41’ 0.5” S 76° 23’ 58.9” W). Samples collected from December 2016 to October 2019. Collection number: CTR-2-46.

Remarks: *Atroporus yasuninensis* was described from basidiomata and rhizomorphs collected over three years in an area of 20 x 10 m^2^ in a temporarily flooded forest. It is characterized by gregarious, medium to big basidiomata (up to 10 cm), centrally stipitate. Pilei infundibuliform with irregular wavy-striate margins, up to 1.5 mm thick. Compared to *A*. *yasuniensis*, *A*. *rufoatratus* and *A*. *diabolicus* have relatively smaller basidiomata with thicker pilei and have been reported to have solitarily habit. Microscopically, *A*. *yasuniensis* has similarities with *A*. *rufoatratus*, with thick-walled distinctive stalks with pointing apex and 1–5 branches present in the trama. Cystidioles shape differs between *A*. *rufoatratus* and *A*. *yasuniensis*, with subulate cystidioles in *A*. *rufoatratus* and ventricose and oblong-like cystidioles in *A*. *yasuniensis*. Basidiospores are similar to other taxa in *Atroporus*; ellipsoid, narrowly ellipsoid, to slightly cylindrical, hyaline, smooth. Rhizomorphs cylindrical to slightly flat, solid, robust, flexible, coriaceous, well-branched, long, bearing a black-reddish cuticle. Rhizomorphs grow abundantly in *A*. *yasuniensis*; however, their development has not been established in *A*. *rufoatratus* and *A*. *diabolicus*.

**Fig 5:**
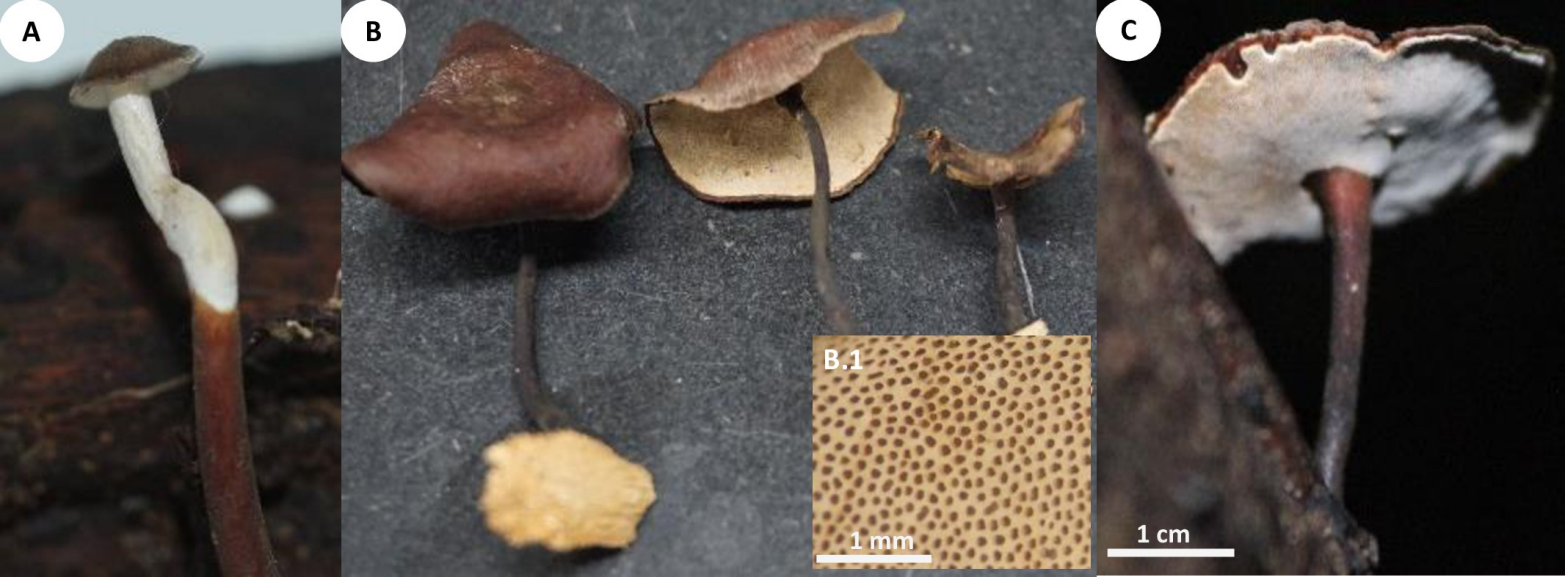
Basidiomata of *Atroporus tagaeri*. **A.** Young basidiome with ongoing melanization of stipe. **B.** Dried basidiomata with fully melanized stipes and shrunken pileus. **B.1** Pore layer. **C.** Fresh basidiome.

**Fig 6:**
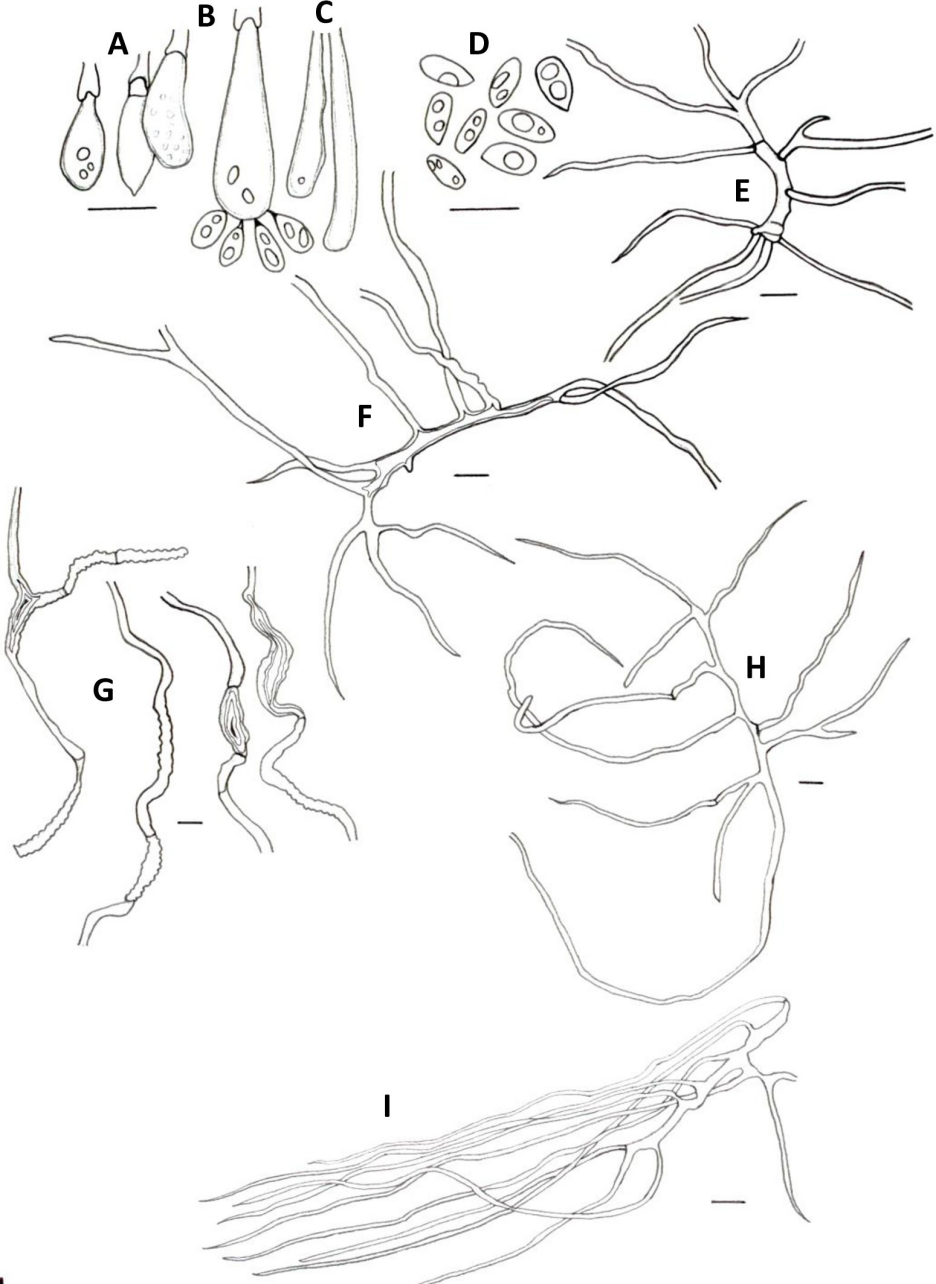
Microscopical features of *A*. *tagaeri*. **A.** Basidioles. **B.** Basidium. **C.** Hyphidia. **D.** Basidiospores. **E-F** Hyphae from trama. **E.** Generative hyphae. **F.** Skeletal-binding hyphae **G-H.** Hyphae from context. **G.** Plectenchymatous thickenings. **H.** Skeletal-binding hyphae. **I.** Skeletal-binding hyphae from stipe. Scale bars = 10 μm.

**2. *Atroporus tagaeri*** Toapanta-Alban, Ordoñez & Blanchette, sp. nov. Figs 5 and 6.

*Typus*. Ecuador, Orellana Province, Yasuní Forest Dynamic Plot. Collected by C E Toapanta-Alban,19 Jul 2017. Collection number: CTR-2-55. Found growing on dead wood of angiosperms.

Mycobank no.: MB 837972

Holotype: Fungarium number: QCAM7412 (QCAM)

    Collection number: CTR-2-55, Toapanta-Alban, July 2017

    GenBank no.: (ITS: MT950129), ((LSU: MT950159, (EF1-α: MW287084), (SSU: MT950205).

Etymology: tagaeri from the Huaorani language (Wao-Terero) in reference to the voluntarily isolated tribe, named after one of their members Tagae. Tribe living in and near Yasuní National Park, Ecuador.

Basidiomata annual, gregarious, centrally stipitate, up to and 4 cm tall. Pileus circular, convex to slightly umbonate with regular margins when young; circular, slightly depressed when old; up to 3 cm in diameter and 0.7 to 1 mm thick (Fig 5).

Pilear surface smooth, glabrous, isabeliline (Munsell’s reference: 0. 7Y/5.2/4.1) with brown vinaceous (Munsell’s reference: 5.OR/2.8/2.0) concentric discolorations near the margin when young; with age dark bay to dark vineaceous (Munsell’s reference: 8. 1R/3.1/6.9; 10. ORP/3.4/4.3. Pilear texture tender when young; coriaceous, robust when old; and rigid, slightly shrunken, when dried. Pore surface white greyish when young; whitish with buff sections (Munsell’s reference: 1.2y/8.1/4.1) when aged; dark buff (Munsell’s reference: 1.2y/8.1/4.1) when dried. Pores circular to slightly angular, 6–9 per mm (ave = 7), 45–90 μm diameter (ave = 68) (Fig 5B1). Dissepiments entire, 30–90 μm (ave = 50). Tubes concolorous with pore surface, uniform not stratified, up to 0.40 mm; IKI+ reaction with tubes turning bright red. Context homogeneous, creamy when fresh; light brown when dried, up to 0.4 mm thick; IKI-. Stipe cylindrical, solid, glabrous, bearing a reddish lacquered cuticle at the base and white near the pileus when young; opaque, dark-reddish to black when aged; up to 3 mm diameter and 1.5–3.5 cm long.

Hyphal system dimitic with generative and skeletal-binding hyphae. In the trama, generative hyphae hyaline, highly branched with thin (2–3 μm) and thick-walled (2–5.5 μm) sections; simple septa and clamp connections. Thin-walled sections along branches and thick-walled sections on the main stem (Fig 6).

Skeletal-binding hyphae found in the trama, context, and stipe. In the trama and context, skeletal-binding hyphae are of the arboriform type, hyaline, thin and thick-walled 1–4 μm, highly branched with simple septa. In context, hyphal modifications are frequent as segments of plectenchymatous thickenings (4–10 μm wide and 39–120 μm long) with simple septa. Skeletal-binding hyphae in the stipe also of the arboriform type, hyaline, 1–4 μm wide with simple septa. The hymenium is composed of paraphysoids structures (cystidioles, basidioles, and hyphidia) and basidia. Hyphidia, smooth, tubular, 3–3.5 μm (ave = 2.7) wide, IKI-. Cystidioles rare, of the ventricose type (10 x 6 μm). Basidioles abundant, clavate 4.3–6.3 μm wide x 10–15 μm length (ave = 6 x 13μm). Basidia, clavate, 4-sterigmata, difficult to find free, (7 x 26 μm). Basidiospores narrowly ellipsoid, thin-walled, hyaline, smooth, IKI-, 5.5–8 μm (ave = 6) x 2.6–4 μm (ave = 3), Q = 1.6–2.12 (ave = 1.90, n = 40).

No rhizomorphs were associated with basidiomata.

Cultural characteristics: No cultures were obtained.

Substrate: Basidiomata emerge from decayed fine woody debris of angiosperms.

Distribution: Known from the Amazon rainforest of Ecuador along ridges and slopes (tierra firme forest).

Specimens examined: Ecuador-Francisco de Orellana Province, Yasuní National Park (0° 41’ 0.5” S 76° 23’ 58.9” W). Samples collected on July 2017. Collection number: CTR-2-55.

Remarks: *Atroporus tagaeri* was described from samples collected in an area of 1m^2^ in terra firme forest. It is characterized by gregarious, small to medium basidiomata (up to 4 cm), centrally stipitate. Pilei circular, slightly depressed with regular margins, up to 3 cm diameter and 1 mm thick. In terms of shape and size, *A*. *tagaeri* is similar to *A*. *rufoatratus*, however, *A*. *tagaeri* has thinner pilei. Hyphal system dimitic, with generative and skeletal-binding hyphae, similar to other taxa in *Atroporus*. The distinctive thick-walled stalks with pointing apex, aid in the description of the genus were not found in the examined specimens. Cystidioles are less frequent in *A*. *tagaeri* than in *A*. *yasuniensis*, *A*. *rufoatratus*, and *A*. *diabolicus*. Plectenchymatous thickenings are abundant in the context and not registered for other taxa in the genus. Basidiospores are similar to other taxa in the genus; narrowly ellipsoid, thin walled, hyaline, IKI-. No rhizomorphs found associated with basidiomata.

***Neodictyopus*** Palacio, Robledo, Reck & Drechsler-Santos, PLoS ONE 12(10), e0186183, (2017)

Type species: *Neodictyopus atlanticae*.

Type specimen: Brazil, Santa Catarina, Santo Amaro de Imperatriz, 15 Nov 2013, ER, Drechsler-Santos DS1285 (FLOR 60309)

*Neodictyopus* was proposed by Palacio, Robledo, Reck & Drechsler-Santos (2017) after decrypting the *Polyporus dictyopus* species complex. The genus was proposed using specimens collected in Brazil-Mato Groso morphologically linked to the type specimen from Chile-Juan Fernández archipelago. *Neodictyopus* was established as a neotropical genus, but it could be presumably present pantropically. Palacio et al. (2017), recommended carrying out surveys and collections from the type locality in order to define the circumscription and distribution.

The genus is also part of the “Melanopus group” since it shares the macromorphological similarities previously described.

Description: “Basidiomata annual, lateral to eccentrically, rarely centrally stipitate; pileus reniform to flabelliform; pilear surface glabrous, radially striate, dark reddish brown; margin irregular, wavy and lobed to decurved and entire. Pores circular. Context homogeneous, yellow to light brown. Stipe cylindrical, solid, reticulated to longitudinally striate, bearing a black cuticle. Hyphal system dimitic; generative hyphae clamped, hyaline, thin-walled. Branched skeletal-binding hyphae dominating, arboriform, hyaline, IKI- to slightly dextrinoid (only in mass) in the trama of the tubes. Basidia clavate, 4-sterigmate. Subcylindrical to bacilliform, thin-walled, smooth, hyaline, IKI-.” [17].

Remarks: Basidiomata lateral to eccentrically stipitate; pileus reniform to spatulate. Hyphal system dimitic, with clamped generative hyphae and skeletal-binding hyphae of the arboriform type, slightly dextrinoid (in mass only) in the trama. Basidiospores subcylindrical to bacilliform. Distributed along the Neotropics, however, might be pantropical. All species grow on dead wood producing white rot on the substrate. *Neodictyopus* is morphologically similar to taxa within the *Polyporus* and *Atroporus* genera. However, *Neodictyopus* differs from the type species of *Polyporus* (*P*. *tuberaster*) in the texture of the pileus and from *Atroporus* by the spore shape and the strong dextrinoid reaction in the skeletal-binding hyphae in the trama proper of *Atroporus*.

**Fig 7:**
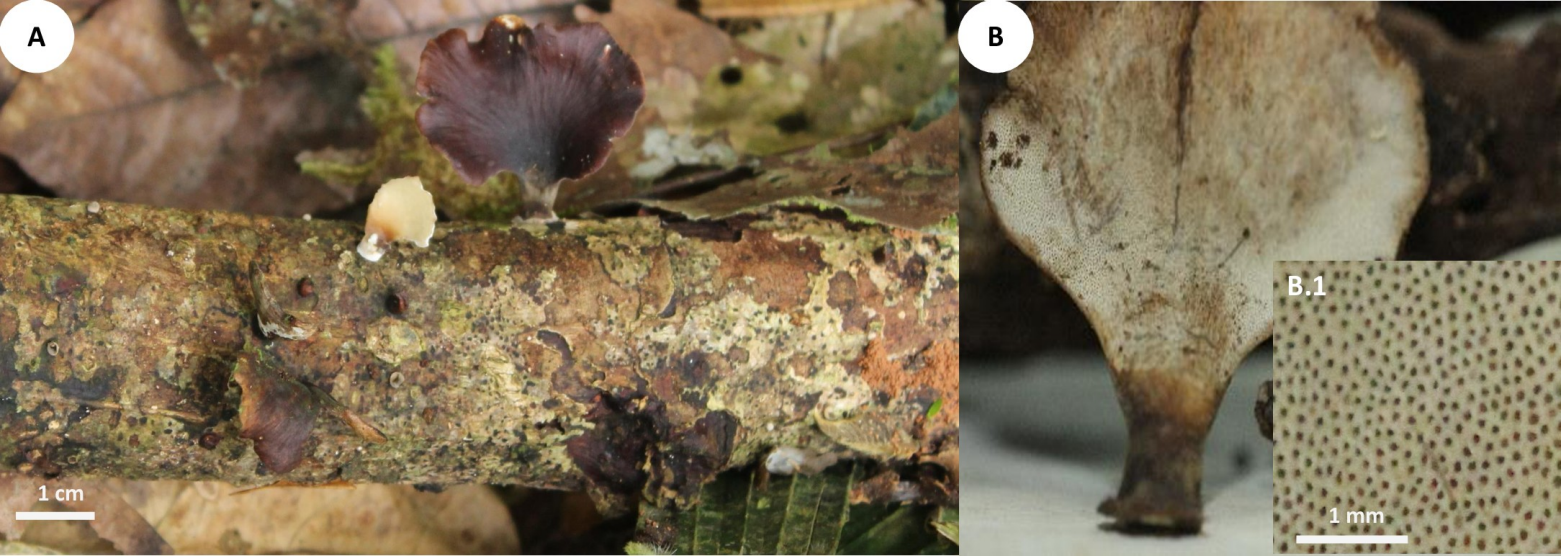
Basidiomata of *Neodictyopus sylvaticus*. **A.** Gregarious habit of basidiomata on substrate. **B.** Hymenium layer. **B.1** Pore layer.

**Fig 8:**
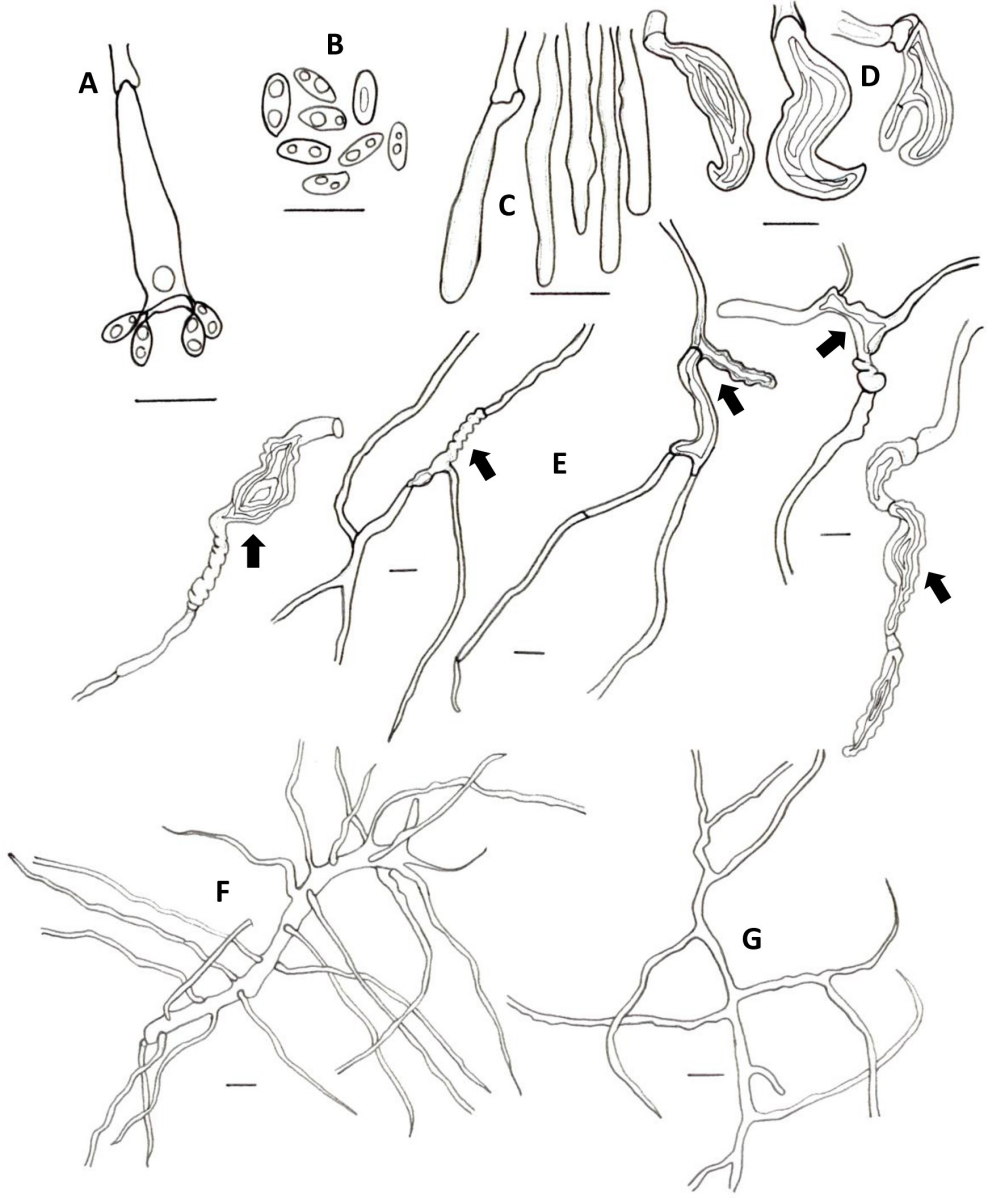
Microscopical features of *N*. *sylvaticus*. **A.** Basidium. **B.** Basidiospores. **C.** Hyphidia. **D.** Tramal setae. **E.** Hyphae from trama with segments of plectenchymatous thickenings. **F-G.** Hyphae from context. **F.** Highly ramified skeletal-binding hyphae. **G.** Thin-walled sections of skeletal-binding hyphae from context. Scale bars = 10 μm.

**3. *Neodictyopus sylvaticus*** Toapanta-Alban, Ordoñez & Blanchette, sp. nov. Figs 7 and 8.

*Typus*. Ecuador, Orellana Province, Yasuní Forest Dynamic Plot. Collected by C E Toapanta-Alban, 22 Jul 2017. Collection number: CTR-2-60. Found growing on dead wood of angiosperms.

Mycobank no.: MB 837973

Holotype: Fungarium number: QCAM7418 (QCAM)

    Collection number: CTR-2-60, Toapanta-Alban, 22 Jul 2017

    GenBank no.: (ITS: MT950155), (LSU: MT950183, (EF1-α: MW287085), (RPB1: MW287111), (SSU: MT950229).

Etymology: sylvaticus from Latin sylva (“forest”), in reference to the geographical region where specimens were found, Amazon rainforest of Ecuador.

Basidiomata annual, gregarious, centrally to eccentrically stipitate, up to and 2.5 cm tall. Pileus reniform to flabelliform with regular to irregular wavy margins; up to 2.3 cm in diameter and 0.83–1.6 mm thick (Fig 7).

Pilear surface smooth, glabrous, yellow-creamy to pale luteous (Munsell’s reference: 2. 4Y/8.5/7.0) when young; sienna, dark bay to dark vinaceous (Munsell’s reference: 6. OYR/5.1/10.8; 8. 1R/3.1/6.9; 10. ORP/3.4/4.3) when old and dried. Pilear texture coriaceous when fresh and slightly shrunken and rigid when aged. Pore surface light creamy when fresh; creamy to light-brown with pale hazel hues (Munsell’s reference: 9. OYR/5.5/3.7) when dried. Pores circular to slightly angular, 6–9 per mm (ave = 7), 45–90 μm diameter (ave = 70); pores are not evenly formed in the hymenium as some regions lack pores (Fig 7B1). Dissepiments entire, 45–110 μm (ave = 80). Tubes concolorous with pore surface, uniform not stratified, 0.30–0.40 mm; IKI+ reaction with tubes turning bright red. Context homogeneous, light to dark brown, up to 1 mm thick; IKI-. Stipe flat, robust, solid, glabrous, bearing black cuticle, up to 5 mm diameter, 2 mm thick, and 5 mm long.

Hyphal system dimitic with generative and skeletal-binding hyphae. In the trama, generative hyphae, hyaline, highly branched with thick and thin-walled sections; thicker sections, slightly rounded, 3–5.5 μm along the main stem and thinner sections 2–3 μm on branches; simple septa and clamp connections (Fig 8).

Skeletal-binding hyphae of the arboriform type, abundant, in trama, context, and stipe, hyaline, thin and thick-walled 1.5–4.5 μm; simple septa. In trama, hyphal modifications are frequent as segments of plectenchymatous thickenings (3–18 μm wide and 19.5–105.5 μm long) with simple septa. Tramal setae found embedded in the tubes, rare, of the ventricose type, slightly hooked, thick-walled, (20–30 μm long x 6–12 μm wide). The hymenium is composed of hyphidia and basidia. Hyphidia, smooth, tubular, 2–5 μm (ave = 3.5) wide. Basidia, rare, narrowly clavate, 4-sterigmata, (6 x 26 μm). Basidiospores narrowly ellipsoid to cylindrical, thin-walled, hyaline, smooth, IKI-, 5–7 μm (ave = 6) x 2.5–3.5 μm (ave = 2.7), Q = 1.8–2.5 (ave = 2.15, n = 40).

No rhizomorphs were found associated with basidiomata.

Cultural characteristics: No cultures were obtained.

Substrate: Basidiomata emerge from decayed fine woody debris of angiosperms.

Distribution: Known from the Amazon rainforest of Ecuador along ridges and slopes (tierra firme forest).

Specimens examined: Ecuador-Francisco de Orellana province, Yasuní National Park (0° 41’ 0.5” S 76° 23’ 58.9” W). Samples collected on July 2017. Collection number: CTR-2-60.

Remarks: *Neodictyopus sylvaticus* was described from samples collected in an area of 1m^2^ in terra firme forest. It is characterized by gregarious, small basidiomata (up to 3 cm), centrally to eccentrically stipitate. Pilei reniform to flabelliform with regular to irregular wavy margins, up to 2.5 cm diameter and 1.5 mm thick. In terms of shape, *N*. *sylvaticus* is similar to *N*. *dictyopus*, however, *N*. *sylvaticus* has smaller basidiomata with thinner pilei. Stipe is short, flat, solid, robust, which differ from the other taxa in the genus, described to have circular stipes. Hyphal system dimitic, with generative and skeletal-binding hyphae. Generative hyphae are distinctive from other taxa in the genus. These are highly branched, thin and thick-walled, slightly rounded along the main stem. In trama, hyphal modification as plectenchymatous thickenings are common. Such hyphal modifications also found in the context of *A*. *tagaeri*, but not reported in any other taxa in *Neodictyopus*. Tramal setae embedded in tubes are very rare, reported to *N*. *sylvaticus* but not in other taxa in *Neodictyopus* or allied genera. Basidiospores narrowly ellipsoid to cylindrical. Rhizomorphs have been reported for *P*. *dictyopus*, however, no rhizomorphs were found associated with *N*. *sylvaticus*.

### Herbarium sample examined

**4. *Neodictyopus* sp.**

**Fig 9:**
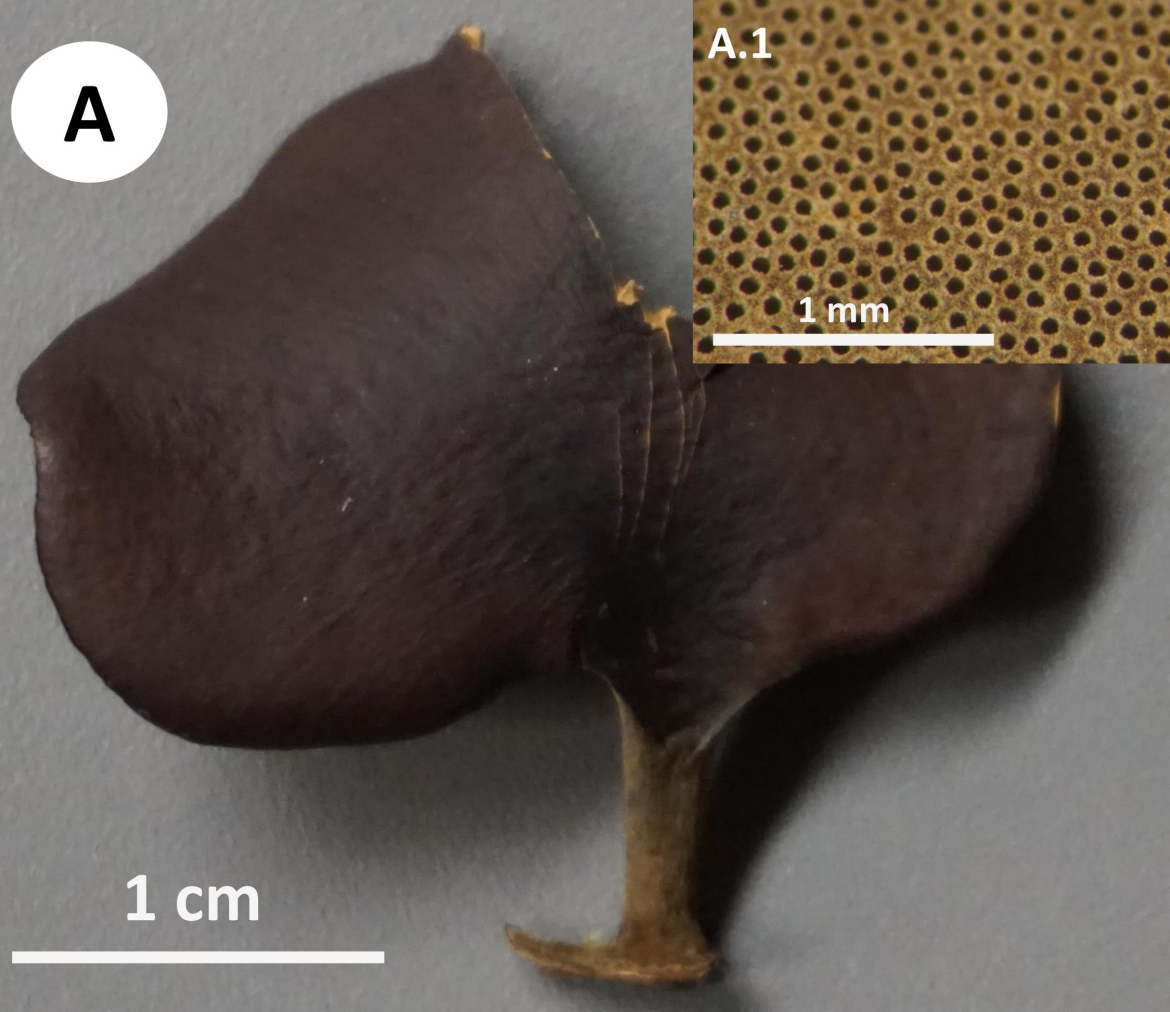
Basidiomata of *Neodictyopus* sp. Ec-97. **A.** Dried basidiome. **A1** Pore layer.

Collection label: Ecuador, Sucumbios Province, Lago Agrio, Camino Los Salideros. Cuyabeno Wildlife Reserve, 0° 00’ N 76° 10’ W, 250 meters above sea level. Collected by Jean Lodge, 8 May 1993. Collection number: EC-97. Found growing on wood-branches. Fig 9.

Mycobank no.: As species rank was not determined, the sample was not accessioned in Mycobank.

Fungarium number: EC-97 (CFMR), in Center for Forest Mycology Research (Fungi of Napo).

GenBank no.: (ITS: MT950132)

Basidiome annual, eccentrically stipitate, solitary, up to 3 cm tall. Pileus reniform to flabelliform with regular margins; up to 3 cm in diameter and 1 mm thick. Pilear surface smooth, glabrous, dark bay to dark vinaceous (Munsell’s reference: 6. OYR/5.1/10.8; 8. 1R/3.1/6.9; 10. ORP/3.4/4.3) (Fig 9).

Pilear texture rigid. Pore surface greyish to dark brown. Pores circular, 7–11 per mm (ave = 9), 41–62 μm diameter (ave = 51). Dissepiments entire, 39–81 μm (ave = 56). Tubes concolorous with pore surface, up to 0.30 mm thick, IKI+. Context homogeneous, light to dark brown, up to 0.7 mm thick; IKI-. Stipe circular, solid, glabrous, bearing a black cuticle, up to 2.5 mm diameter and 8 mm long.

Hyphal system dimitic with generative and skeletal-binding hyphae. Generative hyphae, hyaline, highly branched, thin-walled 1–3 μm, simple septa and clamp connections. In trama, thick-walled distinctive stalks with slightly rounded pointing edges and 1–3 branches located from the middle portion to the tips, 50–81 μm (ave = 62.6 μm) long and 2–4.5 μm (ave = 3.2 μm) wide with a basal clamp connection. Skeletal-binding hyphae of the arboriform type found in trama, context and stipe, hyaline, thin and thick walled; thin-walled sections 1–3 μm along branches and thick-walled sections along the main stem 2–7 μm. In context, hyphal modifications are frequent with segments of plectenchymatous thickenings 40–145.5 μm (ave = 68 μm) long and 3.6–8 μm (ave = 6.4 μm) wide; clamp connections observe where ramifications start. Hymenium composed of paraphysiods structures (cystidioles, basidioles, and hyphidia). Hyphidia, smooth, tubular, thin-walled. Cystidioles of the ventricose type 8–18 μm x 3–6.5 μm. Basidioles, clavate, 7.5–12.7 μm x 3.6–5.8 μm. Basidia not found. Basidiospores not found, however, measurements found on collection label are: 5–7 x 2.5–3.5 μm, Q = 1.6–2.3 (ave = 1.9); ellipsoid to narrowly ellipsoid, slightly cylindrical.

Cultural characteristics: EC-97 Isolate was provided by the USDA Forest Products Laboratory, Center for Forest Mycology Research, Madison, Wisconsin, USA. Cultures were successfully maintained in MYA only; slow growth rate, 10 mm during a month of growth. Cultures highly melanized with, rare formation of aerial mycelium. With time, aerial mycelium forms a dark coriaceous, scar-like tissue, with concentric black discolorations, very tough and difficult to cut.

Substrate: wood-branches.

Distribution: Known from the Amazon rainforest of Ecuador.

Specimen examined: Ecuador-Sucumbíos Province, Camino Los Salideros, Cuyabeno Wildlife Reserve, 0° 00’ N 76° 10’ W, 250 m.a.s.l. Collected by Jean Lodge, 8 May 1993. Collection number: EC-97.

Remarks: The herbarium sample examined was collected in Cuyabeno Wildlife Reserve, Ecuador in 1993. It was previously determined as *P*. *leprieurii*, however, based on macromorphological characteristics and phylogenetic analysis we determined this sample belongs to *Neodictyopus*. This specimen is characterized by basidiome, eccentrically stipitate, solitary; pile reniform, dark vinaceous. Stipe, circular, short, bearing a black cuticle. Macromorphological characteristics in terms of shape and color are similar to *N*. *dictyopus*, however it has a smaller basidiome. Cystidioles of the ventricose type, which are different in shape to the ones present in *N*. *atlanticae* and *N*. *dictyopus* with subulate cystidioles. Thick-walled distinctive stalks with rounded pointing-edges and 1–3 branches are present in trama, similar structures present in most species of *Atroporus*. In context, plectenchymatous thickenings are abundant. The presence of rhizomorphs was not annotated on the label, however, *P*. *dictyopus*, which is closely related to this taxon, has been reported to develop rhizomorphs.

***Polyporus*** P. Micheli ex Adans., Familles des plantes 2: 10 (1763)

Type species: *Polyporus tuberaster* (Jacq. Ex Pers.) F., Systema Mycologicum 1:347 (1821)

Type specimen: Plate 71, Fig 1 in Micheli (1729), was selected as lectotype in Ryvarden (1991)

*Polyporus* accommodates many morphologically heterogeneous species. Taxa within the genus has been accommodated in six infrageneric groups. The genus has undergone important taxonomical changes since it first was proposed. Initial changes were based on macro and micromorphological observations [34]. The later changes were made with the development of molecular biological techniques that allowed to identify the phylogenetic relationships within the taxa under *Polyporus* [15–18]. More taxonomic changes are likely to occur as more sophisticated phylogenetic studies are available.

Description: The type concept followed in this study refers to species with stipitate basidiomata growing on wood or arising from a sclerotium. Basidiomata can be annual or biannual, centrally, eccentrically, or laterally stipitate. Pilei can be circular to dimidiate, convex to infundibuliform; pilear surface smooth to scaly, glabrous to finely tomentose, white to deep brown or black; pilear texture tough when fresh, leathery, or brittle when dry. Pore surface white to creamy, or dark brown when dry. Pores entire, circular to angular, small to large, decurrent or not on the stipe. Stipe creamy to black, glabrous to finely tomentose, with or without a cuticle, smooth to longitudinally wrinkled, in some. Hyphal system dimitic, with arboriform vegetative hyphae; generative hyphae hyaline with clamp connections and simple septa and skeletal-binding hyphae hyaline to brown, solid, or with a lumen. Cystidia none. Hyphal pegs present or absent. Basidia clavate, 4-sterigmate. Basidiospores cylindrical to subellipsoid, straight to slightly bent, thin-walled, smooth, hyaline, IKI-. Substrate, woody debris of angiosperms, conifers, or developing from sclerotia buried in the ground. Species are mostly saprophytes and rarely parasites, causing white rot on wood [4].

Remarks: *Polyporus* is a heterogeneous genus, cosmopolitan with ecologically diverse species. The description provided above is almost universally accepted. It focused on stipitate basidiomata with a dimitic hyphal system of the arboriform type and skeletal-binding hyphae. Basidiospores cylindrical to subellipsoid, IKI-. Substrate, woody debris of angiosperms, conifers, or developing from sclerotia. Allied genera sharing some macro and micromorphological characteristics are *Echinochaete*, *Favolus*, *Atroporus*, and *Neodictyopus*. Such characteristics include, stipitate basidiomata; hyphal system dimitic; basidiospores cylindrical to subellipsoid, smooth, hyaline. *Echinochaete* differs from *Polyporus* for having setae-like, dark brown organs, partly in the dissepiments, partly on the pileus [4, 35].

### Herbarium sample examined

**5. *Polyporus leprieurii*** Mont.

**Fig 10:**
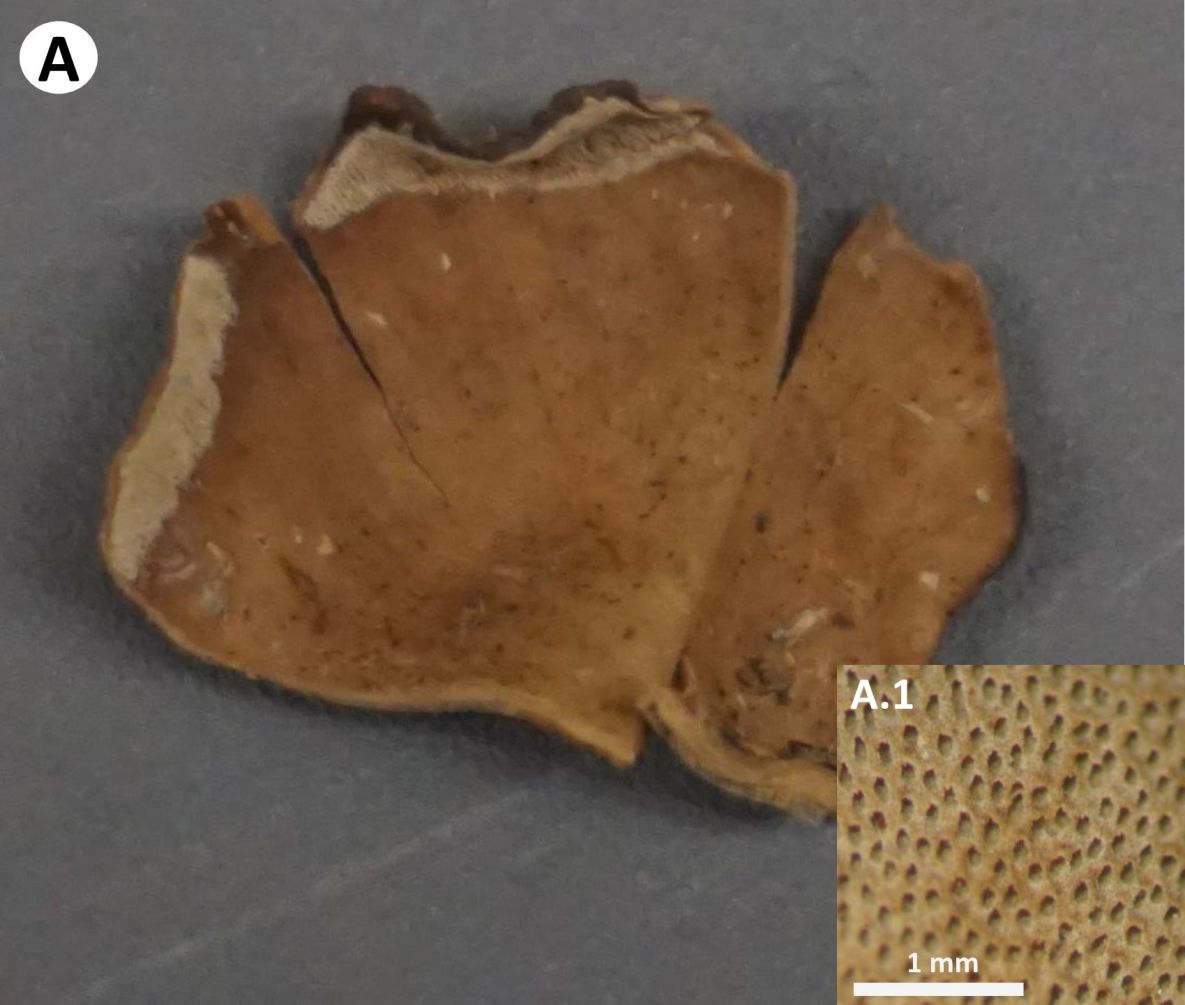
Basidiomata of *Polyporus leprieurii*, BZ-958. **A.** Dried basidiome. **A1** Pore layer.

Collection label: Cayo District-Belize, Maya Mts., Northern foothills, Blue hole National Park on Hummingbird Hwy. (Hummingbird Loop trail). Collection number: LR44178, Collected by Leif Ryvarden, 15 Nov 2001. Fig 10.

Fungarium number: BL-958 (CFMR), in Center for Forest Mycology Research (Fungi of Belize).

GenBank no.: (ITS: MT950154)

Etymology: leprieurii was used after François Mathias René Leprieur, a French pharmacist and naturalist established in Cayenne-French Guiana.

Basidiome annual to biannual, centrally to laterally stipitate (Fig 10).

Pileus reniform to flabelliform with regular margins; 0.5–0.9 mm thick. Pilear surface and texture, smooth, glabrous, rigid; ochraceous, brick, sienna, umber (Munsell’s reference: 10. OR/5.6/6.5; 6. OYR/5.1/10.8; 7. Oyr/3.7/5.8) when dry. Pore surface light brown to pinkish when dried. Pores circular to angular, slightly elongated near margins, 7–11 per mm (ave = 8), 64–90 μm diameter (ave = 80). Dissepiments entire, 25.5–60 μm (ave = 42). Tubes concolorous with pore surface, up to 0.30 mm thick, IKI+. Context homogeneous, light brown, up to 0.63 mm thick; IKI- reaction. Stipe circular, solid, glabrous, bearing a black cuticle, up to 1.5 mm diameter.

Hyphal system dimitic with generative and skeletal-binding hyphae. In the trama, generative hyphae, hyaline, highly branched, thin-walled 1–2.5 μm, simple septa and clamp connections. Skeletal-binding hyphae of the arboriform type, abundant in trama and context, thin and thick walled; thin-walled 1–2.5 μm along branches and thick-walled along the main stem 2.5–4.5 μm. Hymenium composed of paraphysiods structures (basidioles, and hyphidia) and basidia. Hyphidia tubular, smooth, thin-walled, 3.4–6 μm. Basidioles abundant, clavate, 7.5–12.7 x 3.6–5.8 μm. Basidia clavate, four-esterigmate, 20–30 (ave = 21) μm x 4.5–6 (ave = 5) μm. Basidiospores ellipsoid to narrowly ellipsoid, thin-walled, hyaline, smooth, IKI-, 4.5–6.6 (ave = 5.5) μm x 2–3.3 (ave = 2.7) μm, Q = 1.6–2.3 (ave = 2)

Substrate: deciduous wood, mixed broadleaf.

Distribution: Tropical to subtropical species in America and Eastern Asia [Nunez & Ryvarden, 1995].

Specimen examined: Cayo District-Belize, Maya Mts., Northern foothills, Blue hole National Park on Hummingbird Hwy. (Hummingbird Loop trail), 17° 8’ 51” N 88° 40’ 00” W. Collected by Leif Ryvarden, 15 Nov 2001.

Remarks: The species was described by Camille Montagne in 1840 while describing other taxa from French Guiana. *P*. *leprieurii* share macromorphological characteristics with members of the “Melanopus group”. The specimen examined was from the Center for Forest Mycology Research, Wisconsin, was determined as *P*. *leprieurii* by Leif Ryvarden in 2001. It is a partial specimen cut into three pieces; size cannot be determined. Pilear surface smooth, glabrous, ochraceous; rigid texture as dried. Pore surface light brown to pinkish when dry. Pores circular to angular. Stipe solid, glabrous, bearing a black cuticle. Basidiospores ellipsoid to narrowly ellipsoid. Hyphal system dimitic with generative and skeletal-binding hyphae. The production of rhizomorphs is well documented in this taxon; however, their presence was not established on the collection label. *P*. *leprieurii* is known to be distributed in tropical and subtropical areas in America and East Asia. According to “The Global Fungal List”, most records are in Brazil while others are in the Americas (North, Central, and South America), the Caribbean region, Africa (Congo, Cameroon), Northern Asia (Russia), East Asia (China), South and Southeast Asia (India, Sri Lanka, Singapore), and Oceania (Australia, Samoa, Papa New Guinea). Obligated synonyms are *P*. *hemicapnodes* Berk. & Broome, *P*. *pusillus* Rostr., *P*. *calyculus* Pat., & Gaillard, *P*. *savoyanus* Pat., Revue, *P*. *subelegans* Murrill, *P*. *tephromelas* Mont., and *P*. *atripes* Rostr. In terms of shape and size, *P*. *leprieurii* is similar to *Neodictyopus*, however, the color of the pilei differs, with light orange to ochraceous hues in *P*. *leprieurri* and *Neodictyopus* with dark-brown to darker-reddish hues. Presumably, *P*. *leprieurri* could be a species complex that needs further revision to unveil the phylogenetic relationships among specimens in tropical and subtropical regions as macromorphological variations are not obvious between samples in tropical and subtropical regions.

**Fig 11:**
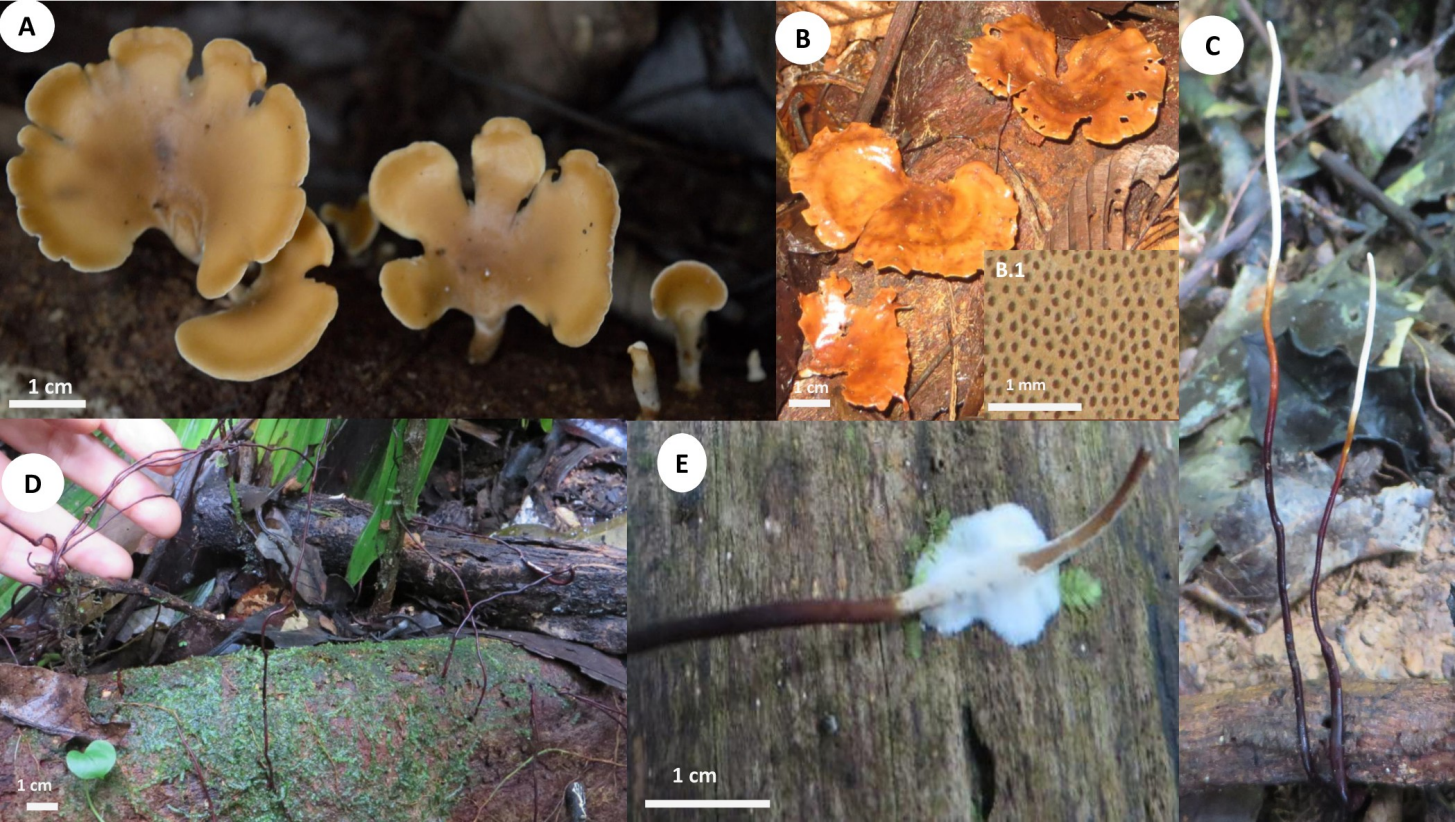
Basidiomata and rhizomorphs of *Polyporus leprieurii* var. *yasuniensis*. **A.** Gregarious habit of basidiomata and primordia. **B.** Aged basidiomata. **B.1.** Pore layer. **C.** Freshly developed rhizomorphs **D.** Aged rhizomorphs emerging from coarse-woody debris. **E.** White rhizomorphic mat formed on new wood substrate.

**Fig 12:**
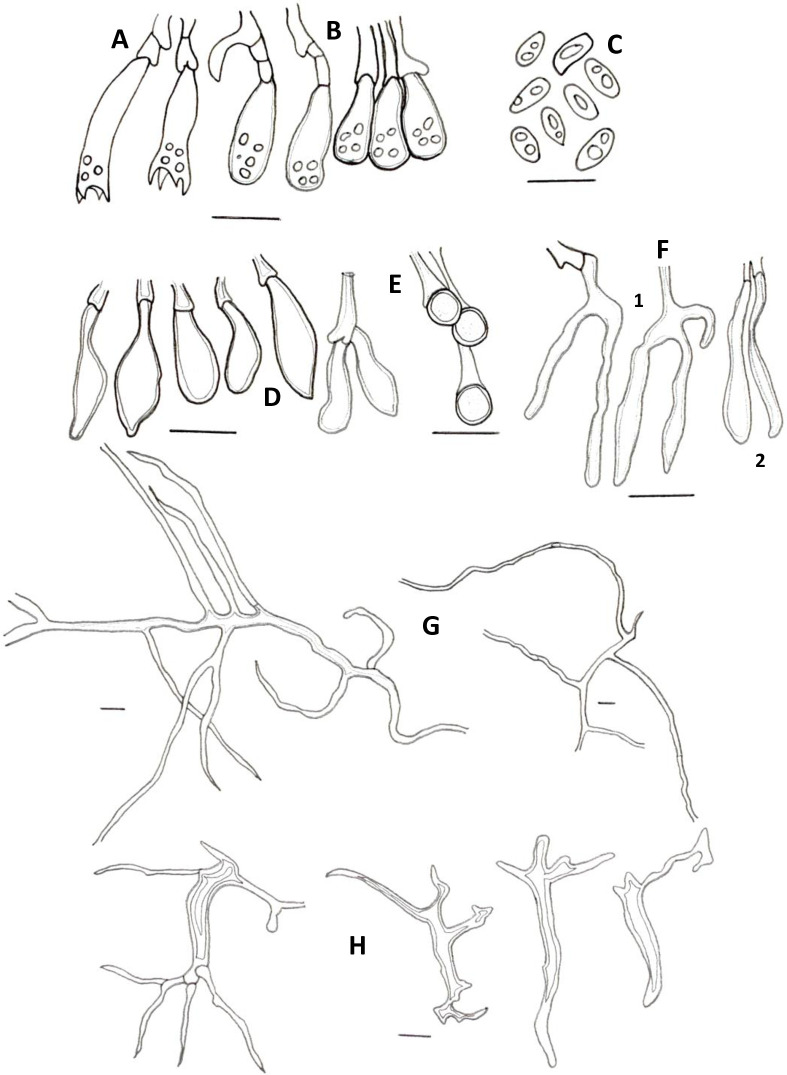
Microscopical features from trama of *P*. *leprieurii* var. *yasuniensis*. **A.** Basidia. **B.** Basidioles. **C.** Basidiospores. **D-E.** Cystidioles. **D.** Cystidioles of the ventricose. **E.** Thick-walled round-like cystidia cells. **F.** Hyphidia, **1.** Dichohyphidia and **2.** Tubular. **G.** Skeletal-binding hyphae. **H.** Thick-walled distinctive stalks of tramal hyphae. Scale bars = 10 μm.

**Fig 13:**
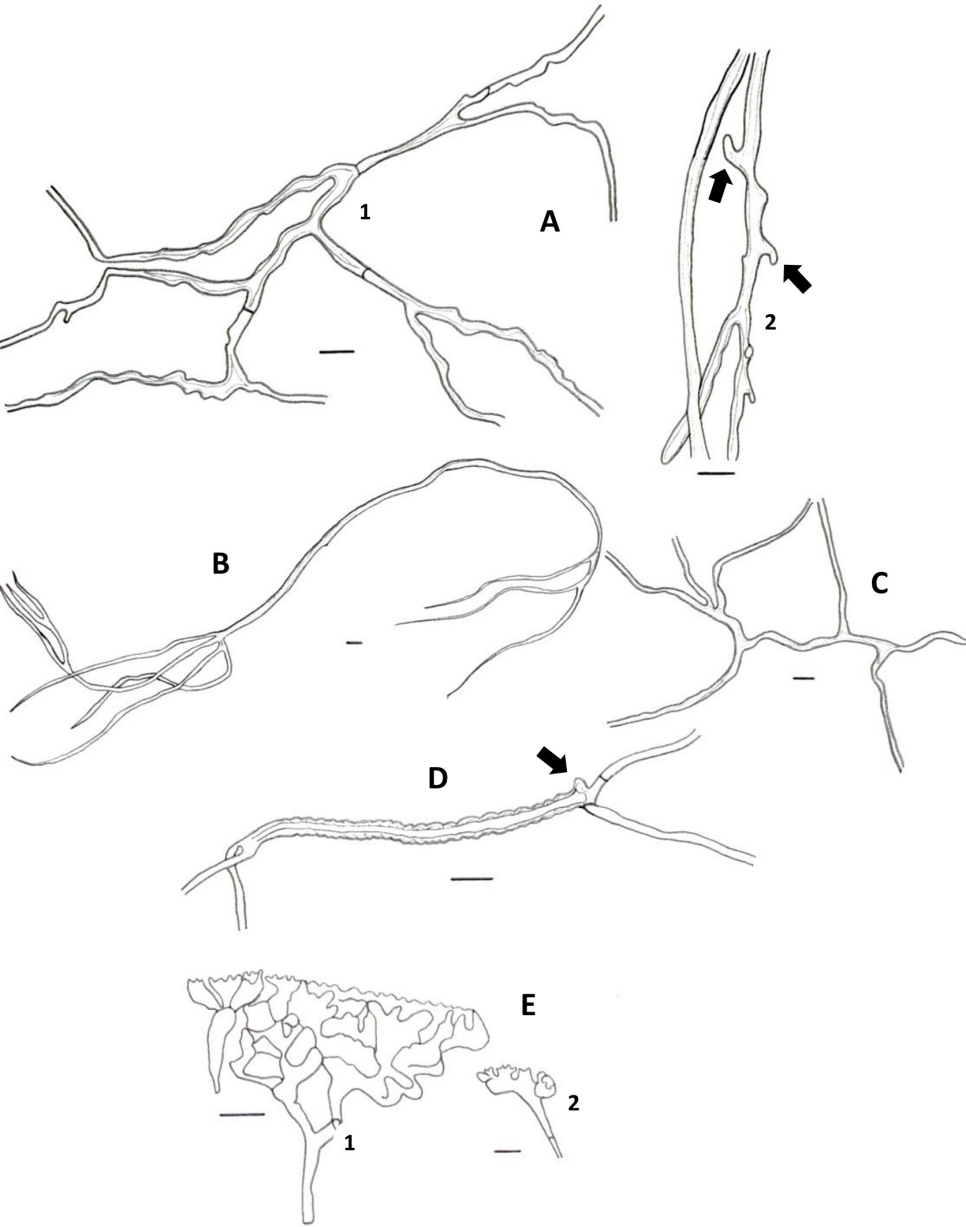
Microscopical features from context, stipe and rhizomorphs of *P*. *leprieurii* var. *yasuniensis*. **A.** Skeletal-binding hyphae from context, **1** Thick-walled sections and **2** Hooked-like sections of hyphae (arrows pointing to hooked sections). **B.** Skeletal-binding hyphae from rhizomorphs. **C.** Skeletal-binding hyphae from stipe **D.** Plectenchymatous thickenings from rhizomorphs (arrow pointing to a clamp connection). **E.** Melanized cuticle cells from stipe, **1** Palisade layer of cells and **2** Individual cuticle cell. Scale bars = 10 μm.

**Fig 14:**
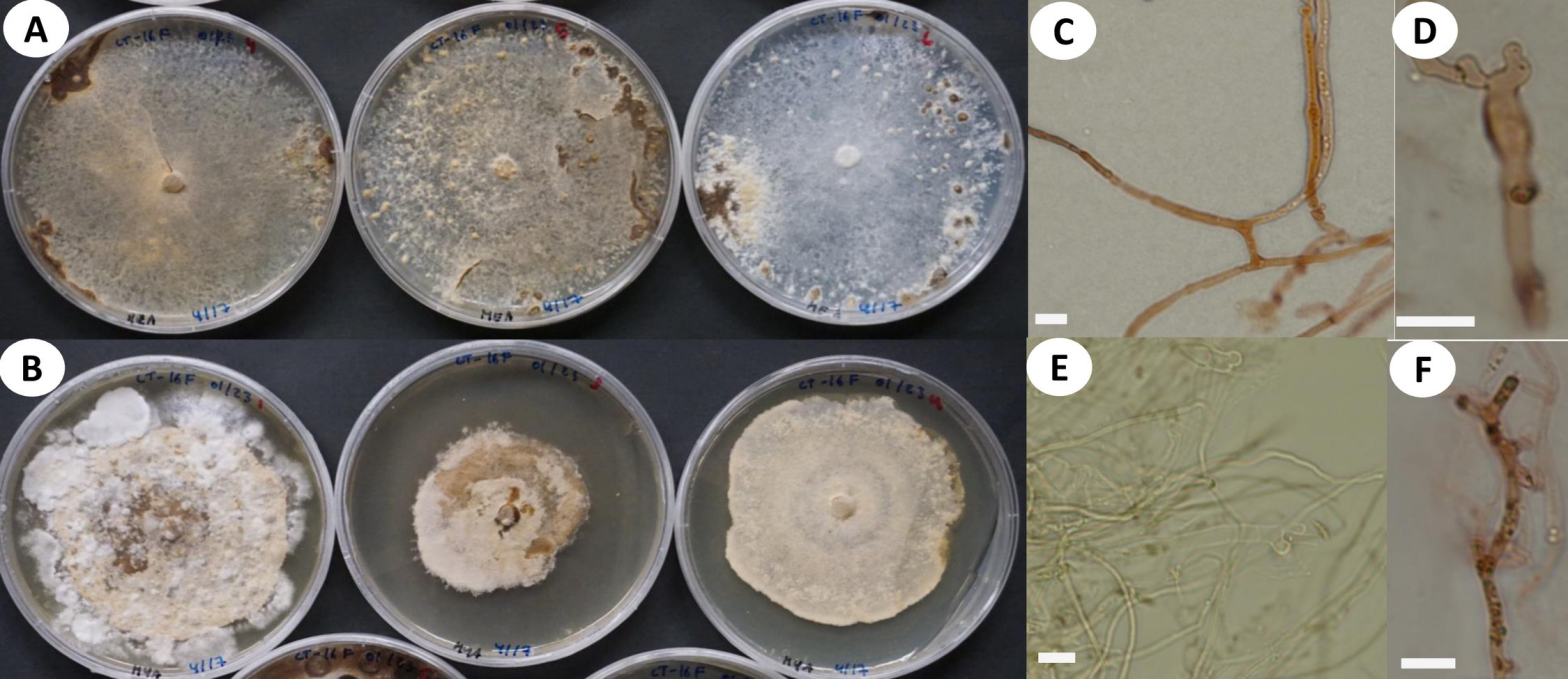
Cultural characteristics and microscopical features of *P*. *leprieurii* var. *yasuniensis*. **A.** Macromorphology of colonies on MEA. **B.** Macromorphology of colonies on MYA. **C.** Slightly branched section of hyphae from white mycelium (Red Congo). **D.** Generative hyphae with clamp connection (Red Congo). **E.** Thin-walled hyphae from white sections of mycelium (Melzer’s reagent). **F.** Highly nucleated section of hyphae (Red Congo). Scales bars = 10 μm.

**6. *Polyporus leprieurii* var. *yasuniensis*** (Mont.) Toapanta-Alban, Ordoñez & Blanchette, var. nov. Figs 11–14.

*Typus*. Ecuador, Orellana Province, Yasuní Forest Dynamic Plot. Collected by C E Toapanta-Alban, 1 Jul 2017. Collection number: CT-16F. Found growing on dead wood of angiosperms.

Mycobank no.: MB 838030

Holotype:    Fungarium number: QCAM7764 (QCAM)

            Collection number: CT-16F, Toapanta-Alban, July 2017

            GenBank no.: (ITS: MT950148), (LSU: MT950177), (EF1-α: MW287101), (RPB1: MW287124), (SSU: MT950223).

Paratypes:    - Fungarium number: QCAM7764 (QCAM)

            Collection number: CT-16F, Toapanta-Alban, October 2019.

            - Fungarium number: QCAM7765 (QCAM)

            Collection number: CTR-1-7.2, Toapanta-Alban, January 2017

            GenBank no.: (ITS: MT95014), (LSU: MT950178), (EF1-α: MW287102), (RPB1: MW287125), (SSU: MT950224).

            - Fungarium number: QCAM7368 (QCAM)

            Collection number: CTR-2-12, Toapanta-Alban, July 2017

            GenBank no.: (ITS: MT950143), (LSU: MT950172), (EF1-α: MW287089), (RPB1: MW287115), (SSU: MT950218).

            - Fungarium number: QCAM7389 (QCAM)

            Collection number: CTR-2-33, Toapanta-Alban, July 2017

            GenBank no.: (ITS: MT950144), (LSU: MT950173), (EF1-α: MW287099), (RPB1: MW287122), (SSU: MT950219).

            - Fungarium number: QCAM7391(QCAM)

            Collection number: CTR-2-35, Toapanta-Alban, July 2017

            GenBank no.: (ITS: MT950145), (LSU: MT950174), (EF1-α: MW287097), (RPB1: MW287121), (SSU: MT950220).

Etymology: yasuniensis (Yasuní = the paradise/God’s creation (from the Wao Terero language use by the Huaorani/Waorani Indigenous People living in the Amazon Rainforest between the Napo and Curaray Rivers in Ecuador)) + ensis (Latin suffix to denote origin). In reference to the site where samples were collected, Yasuní National Park in Ecuador.

Basidiomata annual, gregarious, lateral or eccentrically stipitate, 2–5 cm tall (Fig 11).

Pilei dimidiate, flabelliform or reniform with regular to irregular margins; 1.5–6 cm in diameter and up to 1.3 mm thick. Pilear surface and texture, smooth, glabrous, flexible, coriaceous when fresh and rigid slightly shrunken when dried; yellowish to pale luteous (Munsell’s reference: 2. 4Y/8.5/7.0) when young; ochraceous, brick, sienna, umber (Munsell’s reference: 10. OR/5.6/6.5; 6. OYR/5.1/10.8; 7. Oyr/3.7/5.8) when old. Pore surface white creamy when fresh and light brown pinkish when dried. Pores circular to slightly angular, 6–10 per mm (ave = 8), 40–87 μm (ave = 66). Dissepiments entire, 45–180 μm (ave = 48) (Fig 11 B.1). Tubes concolorous with pore surface, uniform not stratified, up to 0.33 mm (ave = 0.19); IKI+. Context homogeneous, light brown, up to 0.70 mm thick; IKI-. Stipe cylindrical to slightly flat, solid, glabrous, bearing a dark cuticle, brown to reddish when young and dark brown to black when aged; up to 3 mm diameter and 0.60–4 cm long.

Hyphal system dimitic with generative and skeletal-binding hyphae. In the trama, generative hyphae, hyaline, highly branched with thin and thick-walled sections (1–4 μm), with simple septa and clamp connections (Fig 12).

Emerging from the generative hyphae, thick-walled distinctive stalks (up to 7 μm thick and 25–60 μm length) with pointing apexes somewhat rounded to sharp and up to three branches emerging from the middle to the top sections of the stalks; branches thin-walled, 1–3 μm with simple septa. Skeletal-binding hyphae found in the trama, context, stipe, and rhizomorphs (Fig 13).

In the trama and context, skeletal-binding hyphae are of the arboriform type, hyaline, highly branched, thin and thick-walled (1.5–4 μm) with simple septa. Skeletal-binding hyphae in the stipe and rhizomorphs are also of the arboriform type, somewhat less branched, hyaline, thin and thick-walled (1–5 μm) with simple septa. In context and stipe, plectenchymatous thickenings are common, 4.5–11 μm (ave = 6 μm) wide and 17–60 μm (ave = 68 μm) long. In the stipe, generative hyphae can be found holding cuticle cells, which are yellowish to brown, well compacted forming a palisade layer of cells that emerge from thinner hyphae in groups of three or more rounded and lobed cells forming a crown-like shape structure. The hymenium is composed of paraphysoids structures (cystidioles, basidioles, and hyphidia) and basidia. Hyphidia can be tubular and branched (dichohyphidia), hyaline, smooth, 2.5–6 μm (ave = 3) wide, IKI-. Cystidioles can be thick-walled round-like cells (5x5 μm) and of the ventricose type (12–19 x 5–8 μm). Basidia clavate, four-sterigmata, 18–30 μm x 6–10 μm, difficult to find. Basidioles clavate, 8–13 x 4.5–8 μm, abundant in the hymenium. Basidiospores ellipsoid to narrowly ellipsoid, slightly cylindrical, hyaline, thin-walled, smooth, IKI-, 4.5–7 μm (ave = 5.4) x 2.4–3.3 μm (ave = 2.6), Q = 1.50–2.4 (ave = 2).

Rhizomorphs, cylindrical, solid, coriaceous, thin, up to 2 mm diameter and 0.3–1 m long; bearing a black-reddish lacquered cuticle with yellow and white tips when fresh; black and rigid when dried (Fig 12). Rhizomorphs in this taxon are abundant, found in all collection sites (40), distributed on valleys (temporarily flooded forest), slopes, and ridges (terra firme forest). Can be annual to perennial as they can be present for more than four years in colonized areas from 5 m^2^ to 20 m^2^. Rhizomorphs emerge from fine and coarse woody debris, standing and downed dead trees. Often rhizomorphs are found attached to trees and other surfaces to maintain their upward growth. Rhizomorphs attach to new substrates and other rhizomorphs by forming a rhizomorphic mat of white mycelium that turns brown-reddish and becomes darker over time. When attached to other rhizomorphs, they form a complex rhizomorphic network covering living and dead plant material found on the forest floor. Rhizomorphs can accompany basidiomata or be alone. It is rare, but basidiomata can emerge from rhizomorphs.

Cultural characteristics: Cultures were easily obtained from rhizomorphs, basidiomata, and colonized substrate. Initial growth is characterized by white, appressed, silky to cottony mycelium that can turn dark brown to reddish. Colonies can develop crustose melanized tissue at sections or on the whole colony. Some colonies might not be melanized at all. Growth rate, characteristics of the mycelial mat, presence of aerial mycelium, and color of the colony are highly variable.

Grow rate is relatively slow on MEA 29.96–41.45 mm (ave = 33.90) compared to MYA 36.83–42.19 mm (ave = 40.28) after one month of growth (Fig 14).

On MEA, the marginal zone is irregular, usually appressed or submerged in the agar giving the appearance that aerial mycelium is absent in the margin. Aerial mycelium white, floccose, silky, cottony; thick, crustose, coriaceous, brown tissue can be formed over time. The agar can have a glossy brown discoloration and chamois-like appearance when the mycelium is submerged; pseudosclerotial plates are often formed. On MYA, margins of colonies are irregular, generally appressed, and raised near the plate’s wall. Mycelium can be submerged and aerial; when aerial, silky, floccose, cottony white-creamy with dark radial discolorations. Crustose mycelium is often formed on the melanized sections which become tough coriaceous and difficult to cut overtime. Rhizomorphs developed on culture media but only after long periods (6–12 months) of growth apparently after nutrients have been utilized (on MYA only).

Hyphal system is dimitic, with generative hyphae of the arboriform type with thin and thick-walled sections in the white-aerial mycelium and skeletal hyphae in the melanized-crustose sections of mycelium. Generative hyphae, thin-walled sections 1–2.5 μm wide and thick-walled sections 2–6 μm wide; simple septa and clamp connections; IKI-; KOH-. Skeletal hyphae from crustose sections of mycelium are yellowish-brown, similar to hyphae from the cuticle on stipe and rhizomorphs. Skeletal hyphae are arranged in clusters forming a palisade, individual cells 2–8 μm wide, thicker along branches near the melanized tissue, clamp connections abundant.

Substrate: Basidiomata and rhizomorphs emerge from decayed wood: fine and coarse woody debris, small pieces of lignified tissue from the veins of palm leaves, standing and downed dead trees. In laboratory assays, rhizomorphs can emerge from colonized wood of conifers (data not presented here).

Distribution: Known from the Amazon rainforest of Ecuador along valleys, slopes and ridges (temporary flooded and terra firme forests).

Specimens examined: Ecuador, Francisco de Orellana Province, Yasuní National Park (0° 41’ 0.5” S 76° 23’ 58.9” W). Samples collected from December 2016 to October 2019. Collection number: CT-16F, CTR-1-7.2, CTR-2-12, CTR-2-33, and CTR-2-35.

Remarks: The type specimen of *P*. *leprieurii* var. *yasuniensis* was collected in an area of 40 x 30 m^2^ in terra firme forest in Yasuní National Park, Ecuador. This collection site was observed to produce rhizomorphs and basidiomata for over four years. This taxon is very common along the forest floor in terra firme and temporarily flooded forests with collection sites that vary from 0.5–40 m^2^. *P*. *leprieurii* var. *yasuniens*is is characterized by annual basidiomata, lateral or eccentrically stipitate, gregarious. Pilei dimidiate, flabelliform or reniform with regular to irregular margins; thin up to 1 mm thick; coriaceous, flexible when fresh and rigid when dried. Hyphae system dimitic with generative and skeletal-binding hyphae of the arboriform type. Basidiospores ellipsoid to narrowly ellipsoid, slightly cylindrical. Cystidia-like terminal thick-walled rounded structures and abundant cystidioles accompanying basidia and the sterile hyphae in the hymenium. Rhizomorphs common, short to long, thin, flexible, coriaceous, non to highly branched, bearing a black-reddish cuticle. *Neodictyopus*, can be confused with *P*. *leprieurii* (Mont.) and *P*. *leprieurii* var. *yasuniensis*, however they differ in the color of the pilei when basidiomata are aged with white-creamy to orange hues in *P*. *leprieurii* and *P*. *leprieurii* var. *yasuniensis* and reddish to purplish in *Neodictyopus*. Macromorphologically, *P*. *leprieurii* and *P*. *leprieurii* var. *yasuniensis* have non variations. Microscopically, *P*. *leprieurii* (Mont.) and *P*. *leprieurii* var. *yasuniensis* differ in the presence of cystidioles and thick-walled stalks with pointing apex present in the trama of *P*. *leprieurii* var. *yasuniensis*.

**Fig 15:**
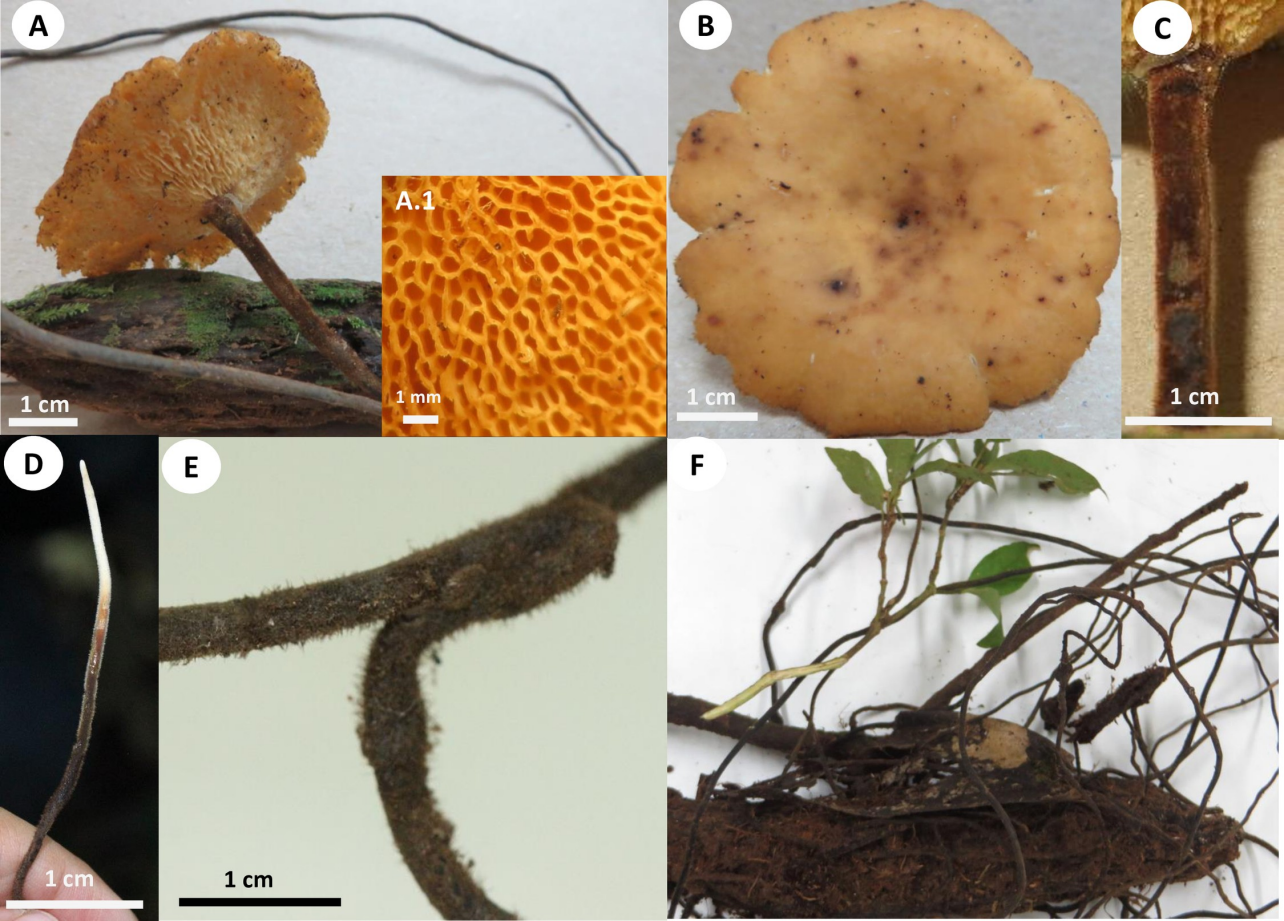
Basidiome and rhizomorphs of *Polyporus taromenane*. **A.** Basidiome and rhizomorphs on substrate **A.1** Pore layer. **B.** Pileus. **C.** Stipe. **D-F.** Rhizomorphs. **D.** Fresh rhizomorph with white tip. **E.** Rhizomorphs with velvety appearance. **F.** Rhizomorphs attached to substrate and living plant material.

**Fig 16:**
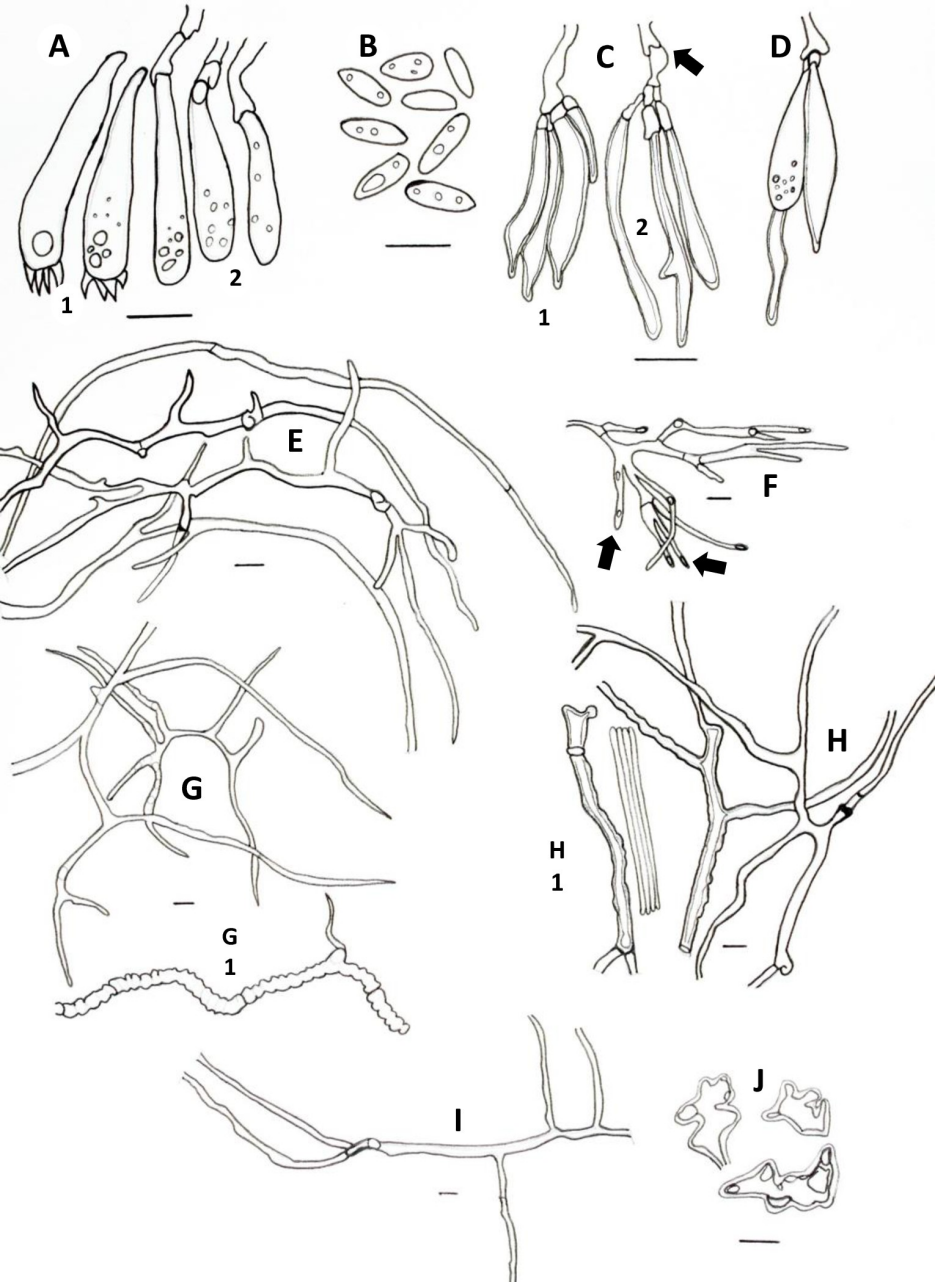
Microscopical features of *P*. *taromenane*. **A.** Basidia (1) and basidioles (2). **B.** Basidiospores. **C.** Paraphysoids structures. **C1** Cystidioles of the ventricose type and **C2** Hyphidia (arrow pointing a clamp connection). **D.** Basidiole, cystidiole of the ventricose type and hyphidia on a generative hypha. **E-F.** Hyphae from trama, with **E.** slightly ramified sections of thin-walled hyphae and **F.** Section of hyphae highly nucleated and highly ramified (arrows pointing on nucleus). **G.** Skeletal-binding hyphae from context**. G.1.** Plectenchymatous thickenings from context. **H.** Skeletal-binding hyphae from stipe. **H.1.** Plectenchymatous thickenings from stipe. **I.** Skeletal-binding hyphae from rhizomorphs. **J.** Individual cuticle cells from stipe. Scale bars = 10 μm.

**Fig 17:**
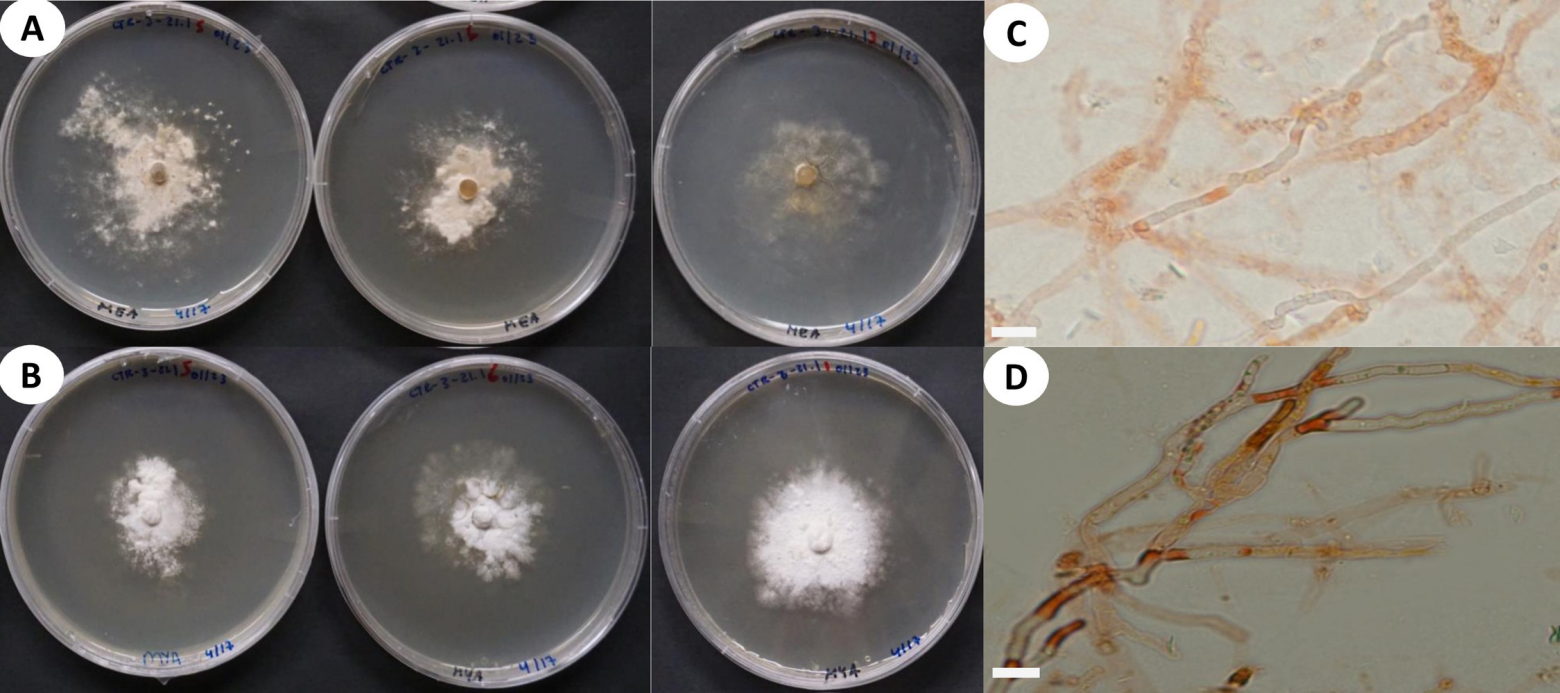
Cultural characteristics and microscopical features of *P*. *taromenane*. **A.** Macromorphology of colonies on MEA. **B.** Macromorphology of colonies on MYA. **C-D** Slightly branched sections of hyphae from white mycelium with simple septa and clamp connections (Red Congo). Scales bars = 10 μm.

**7. *Polyporus taromenane*** Toapanta-Alban, Ordoñez & Blanchette, sp. nov. Figs 15–17.

*Typus*. Ecuador, Orellana Province, Yasuní Forest Dynamic Plot. Collected by C E Toapanta-Alban, 14 Jul 2017. Collection number: CTR-2-42. Growing on dead wood of angiosperms.

Mycobank no.: MB 838030

Holotype:    Fungarium number: QCAM7432 (QCAM)

            Collection number: CTR-2-42, Toapanta-Alban, July 2017

            GenBank no.: (ITS: MT950127), (LSU: MT950158), (EF1-α: MW287087), (RPB1: MW287113), (SSU: MT950203).

            Paratypes:    - Fungarium number: QCAM7766 (QCAM),

                        Collection number: CTR-1-8, Toapanta-Alban, January 2017

            GenBank no.: (ITS: MT950126), (LSU: MT950157), (EF1-α: MW287086), (RPB1: MW287112), (SSU: MT950202).

            - Fungarium number: QCAM7767 (QCAM),

            Collection number: CTR-3-21, December 2018

            GenBank no.: (ITS: MT950128), (EF1-α: MW287088), (RPB1: MW287114), (SSU: MT950204).

Etymology: taromenane from the Huaorani language (Wao-Terero) in reference to the voluntary isolated indigenous clan Taromenane, which are distantly related to the Huaorani people. Meaning unknown.

Basidiome is rare and only one sample was found over three years of sampling at three collection sites with rhizomorphs belonging to this taxon. Basidiome annual, solitary, centrally stipitate, 8 cm tall. Pileus infundibuliform, depressed with regular hirsute margins; 5.5 cm in diameter and 3 mm thick (Fig 15).

Pilear surface smooth, glabrous, pale luteus to amber (Munsell’s reference: 2. 4Y/8.5/7.0 and 4. OY/7.5/8.0). Pilear texture tender when fresh; shrunken and rigid when dried. Pore surface pale luteus (Munsell’s reference: 2. 4Y/8.5/7.0). Pores angular to slightly elongated, 1–2 per mm (ave = 1.5), 0.4–0.9 mm (ave = 0.66) (Fig 15 A.1). Dissepiments entire, 1–3 mm (ave = 2.3). Tubes concolorous with pore surface, not stratified 0.65–1.84 mm (ave = 1.05); IKI+ (slightly red reaction). Context homogeneous, concolorous with pore surface, 0.3–0.4 mm (ave = 0.36) thick; IKI-. Stipe cylindrical, solid, velvety with short uniform white and reddish-brown hairs, bearing a dark-brown cuticle, up to 4.5 cm long and up to 3.5 mm diameter.

Hyphal system dimitic with generative and skeletal-binding hyphae. In the trama, generative hyphae, hyaline, highly branched with thin and thick-walled sections (1–5 μm wide) with simple septa and clamp connections (Fig 16).

Skeletal-binding hyphae found in the trama, context, stipe, and rhizomorphs. In the trama and context, skeletal-binding hyphae are of the arboriform type, hyaline, highly branched, thin and thick-walled (1.5–5 μm wide) with simple septa. Skeletal-binding hyphae in the stipe and rhizomorphs are also of the arboriform type, somewhat less branched, hyaline, thin and thick-walled (1–3.8 μm wide) with simple septa. In context and stipe, plectenchymatous thickenings are common, 4–7 μm (ave = 5.33 μm) wide and 80–147 μm (ave = 90 μm) long; clamp connections observed where ramifications start. In the stipe, generative hyphae hyaline to yellowish, highly branched, thin (1–3.8 μm) and thick-walled (1–3.8 μm). Cuticle cells, which are yellowish to brown, well compacted forming a palisade layer of cells that emerge from thinner hyphae in groups of three or more round to lobed-like cells forming crown-like structure, with simple septa and clamp connections; cuticle 130 μm thick. The hymenium is composed of paraphysoids structures (cystidioles, basidioles, and hyphidia) and basidia. Hyphidia tubular to slightly branched, 3–6 μm (ave = 3) wide, IKI-. Cystidioles of the ventricose type (20–30 x 5–7 μm), rare. Basidia clavate, four-sterigmata, 14–38 μm length (ave = 27) x 5–8 μm wide (ave = 6). Basidioles clavate, 8–13 x 4.5–8 μm, abundant. Basidiospores narrowly ellipsoid to cylindrical, hyaline, smooth, thin-walled, IKI-, 7–10 μm (ave = 8) length x 3–4 μm (ave = 3.5) wide, Q 1.90–3 (ave = 2.40).

Rhizomorphs, cylindrical, solid, coriaceous, thin, up to 3 mm diameter and 2.5 m long; bearing a black-reddish cuticle with white-yellowish tips and small brown hairs; rigid when dried (Fig 15). Rhizomorphs in this taxon are long and not ramified. Rhizomorphs can be annual to perennial as they have been found growing in the same area for up to four years. Rhizomorphs emerge from decayed wood covered with a black melanized cuticle that makes the wood hard to break. Rhizomorphs are not abundant; however, they can be very long and survive longer than the rhizomorphs of *P*. *leprieurii* var. *yasuniensi*s.

Cultural characteristics: Cultures were obtained from colonized substrate. Initial growth is characterized by white, appressed, silky to cottony mycelium that grows relatively slow compared to other fungi in the group. There are small variations regarding grow rate, characteristics of the mat, aerial mycelium and color among colonies growing on different types of media. Colonies can develop melanized tissue, however, it is spotted and appears as small dots on the colony which can be lost overtime.

Growth rate on MEA 8.23–13.13 mm (ave = 11.77) and MYA 7.1–21.31 mm (ave = 10.68) in a month of growth is relatively slow compared to other taxa examined here (Fig 17).

On MEA, the marginal zone is irregular, usually appressed or submerged in the agar giving the appearance that aerial mycelium is absent. Aerial mycelium, white to creamy, compacted, cottony at sections. On MYA, margins of colonies are irregular, generally submerged and slightly appressed. Aerial mycelium, silky, compacted, slightly floccose, white to creamy, but not melanized.

Hyphal system is monomitic, with skeletal-binding hyphae, hyaline, smooth to granular along highly nucleated sections; highly branched; thin and thick-walled (1.7–6.8 μm wide) with clamp connections and simple septa; IKI-; KOH-.

Substrate: Basidiome and rhizomorphs emerge from decay wood (coarse and fine woody debris).

Distribution: Known from the Amazon rainforest of Ecuador along ridges and slopes (terra firme and temporary flooded forests).

Specimens examined: Ecuador, Yasuní National Park (0° 41’ 0.5” S 76° 23’ 58.9” W), Francisco de Orellana Province, samples collected from December 2016 to October 2019. Collection number: CTR-1-8 (rhizomorphs and cultural characteristics), CTR-2-42 (rhizomorphs and Basidiome), CTR-3-21 (rhizomorphs).

Remarks: The type specimen of *P*. *taromenane* was described from a single basidiome collected in October 2019 in an area of 0.5 m^2^ in terra firme forest in Yasuní National Park. The collection site produced rhizomorphs over four years, but only one basidiome was found within that time period. This taxon is somewhat rare compared to *P*. *leprieurii* var. *yasuniensis*, with only three collections within the 50-ha study site. *P*. *taromenane* is characterized by annual basidiomata, central, stipitate, solitary. Pileus circular, infundibuliform, depressed with regular hirsute margins. Big angular to slightly elongated pores. Hyphal system dimitic with generative and skeletal-binding hyphae of the arboriform type. Skeletal-binding hyphae in context and stipe with plectenchymatous thickenings. Basidiospores narrowly ellipsoid to cylindrical. Sterile structures present in the hymenium: ventricose cystidioles are rare; hyphidia common; basidioles clavate, abundant. Rhizomorphs circular, solid, robust, long, somewhat not branched, flexible, coriaceous, bearing a black-reddish cuticle and small brown hairs. *P*. *taromenane* is closely related to *P*. *guianensis* Mont., with this, it shares macromorphological similarities in terms of, pilei color and texture, color and texture of the stipe, pore’s shape and size. Micromorphological differences with *P*. *guianensis*, include the presence of sterile elements in the hymenium and presence of plectenchymatous thickenings in the skeletal-binding hyphae in the context and stipe. There are no reports of rhizomorph development in *P*. *guianensis*, which have most reports in Tropical Asia.

## Discussion

In this work, we contribute to the knowledge of fungal diversity in Yasuní National Park by describing four new species and one new variety, three of them producing aerial rhizomorphs. Yasuní National Park, the biggest natural reserve in Ecuador, is highly biodiverse as well as highly threatened by illegal logging, hydrocarbon and mining projects, as well as land transformation due to oil palm plantations, and large-scale transportation projects [36]. Understanding the true biodiversity of poorly studied groups like fungi, and the role they play in such a biodiverse ecosystem is needed to unveil their ecological significance.

The newly described species *Atroporus yasuniensis*, *Atroporus tagaeri*, *Neodictyopus sylvaticus*, and *Polyporus taromenane*, and the new variety *Polyporus leprieurii* var. *yasuniensis*, were named after Yasuní National Park and the voluntarily isolated indigenous groups (Tagaeri and Taromenane) that live in the area. The results from this study strongly suggests additional taxonomic studies are needed to identify the other very diverse fungi present. A better understanding of the organisms found in the forest will help to enhance conservation efforts and protect this highly biodiverse ecosystem and the people living there.

This taxonomic study was aimed to identify the fungal species producing rhizomorphs that are present in Yasuní National Park. Rhizomorphs are versatile structures and the role they have in colonization, survival, pathogenicity, and ecological function in forest ecosystems has been well documented on subterranean temperate fungal species such as the *Armillaria* species complex [37–40]. However, little is known of the role aerial rhizomorphs have in the tropics or their ecological function in such a diverse forest ecosystem [3, 10, 12, 41, 42]. To contribute to this understanding, we identified and described fungi producing rhizomorphs found on the forest floor. The information presented here will help to better understand the diversity of rhizomorph-forming fungal species and contribute to future ecological studies looking at the role these species play in this ecosystem.

The work took place in one of the 72 permanent forest plots for the long-term study at the Forest Global Earth Observatory (ForestGEO). Permanent forest dynamic plots have become an important starting point for the development of research in tropical ecosystems across a wide range of fields [43]. The little anthropogenic disturbance that occurs in Yasuní Forest Dynamic Plot allowed us to observe and report growth patterns of fungi, including the colonization of new substrates and the production of basidiomata and rhizomorphs across four years of sampling.

Identifying the taxa producing these rhizomorphs can be challenging if basidiomata are not present on the substrate. In addition, for rare species, the likelihood of finding basidiomata and rhizomorphs associated with the species in the same substrate can be very difficult. In the research presented here for taxa associated with the *P*. *leprieurii* group, only 35 collections out of 120 had basidiomata on the substrate, or near the collection sites where rhizomorphs were collected. In the case of *P*. *taromenane*, only three collections among all the surveyed quadrants and transects were found, with only one basidiome present in the same site where rhizomorphs were collected in previous years. In the *Atroporus* group, only *A*. *yasuniensis* has been found to develop large complex systems of rhizomorphs that are well distributed along the forest floor. Basidiomata for this species were only found after the third year of sampling in the area. Basidiomata of other species in the *Atroporus* genus were collected in similar areas, but rhizomorphs associated with them were not found. This exemplifies the challenge mycologists face while linking rhizomorphs to specific taxa, especially when surveys are not repeated in the same site.

The type of rhizomorphs found associated with the taxa studied share some similar morphological features, such as upward growth, coriaceous texture, bearing a dark cuticle, formation of a rhizomorphic/mycelial mat to attach to substrates, and anastomosis of rhizomorphs to form a complex network. However, in the case of *P*. *taromenane*, rhizomorphs in addition to having a black cuticle also have small hairs that give rhizomorphs a velvety appearance and distinguish it from the other taxa in the “Melanopus group”. Phylogenetically, *P*. *taromenane* is quite distance, and we hypothesize there may be other related species in this clade.

Within the *Polyporus* genus and allied genera, rhizomorphs have been reported to be formed as a response to environmental stress [4]. In our study, rhizomorphs were found growing along ridges, slopes (terra firme forest), and valleys (temporarily flooded forests), ecosystems that are exposed to seasonal droughts and exceeding wet conditions. In both ecosystems, the production of rhizomorphs in areas exceeding the 5 m^2^ of the colonized substrate was vast and continuously developed during the four years of sampling. It appears that the establishment of rhizomorphs allows these fungi to survive in areas prone to environmental stresses linked to excessive or limited water availability, and/or to be formed as a strategy to find new substrates and obtain new sources of nutrients. This role of rhizomorph utility was supported by our observation during the cultural characterization assays when rhizomorph development occurred rarely on more than six-months old MYA cultures. However, the influence of water stress and scarce nutrients on the substrate needs further examination to establish the exact influence on the rhizomorph formation within the species in the “Melanopus group”. In addition to the stress response, we believe rhizomorphs provide these species with mechanisms that allow them to prevail in harsh environmental conditions including floods, desiccation during dry periods, and damage from UV light. In addition, melanized rhizomorphs appear to restrict insect predation. This has also been observed in rhizomorphic fungi from temperate forests [37–40, 44]. The complete biological significance of rhizomorphs in the “Melanopus group” should be studied in greater detail to better elucidate their important role in the ecosystem.

The taxa examined in this study are part of the artificially designated “Melanopus group”, within the Polyporaceae family, many of them previously classified as *Polyporus* P. Micheli ex Adans [4]. The group has undergone important recategorization of its taxa since phylogenetics became a tool in taxonomic studies [14–16]. Such studies helped to identify the polyphyletic origin of taxa in the group. The new taxa describe in this study were proposed by phylogenetic differences using reference sequences of closely related taxa.

The “leprieurii” clade appears to be a species complex with taxa morphologically more similar in the neotropics, sharing macro and micromorphological similarities with the original description of the type specimen of *P*. *leprieurii* Mont. These include the position of the stipe, color of the pilei, size of the pores, shape and size of basidiospores, and the presence of rhizomorphs. In the absence of the type specimen, an herbarium sample (BZ-958) from Belize and a reference sequence from Costa Rica were used to compare the phylogenetic and morphological similarities between the samples from Ecuador and specimens from other locations. Samples from Belize and Costa Rica were phylogenetically similar to each other, but distant to the specimens from Ecuador. Micromorphologically, the specimen from Belize was more similar to the type description, as both lack cystidia-like cells, but other hyphal descriptions are similar. The new variety, *P*. *leprieurii* var. *yasuniensis* differs from the type specimen and herbarium sample since it had cystidioles of the ventricose type, rounded cystidia-like cells, and distinctive thick-walled stalks with pointed apex and 1–3 branches accompanying the hyphae in the trama. These distinctive hyphal characteristics have been described and used as diagnostic of *Atroporus* [17]. The specimens described as *P*. *leprieurii* followed the same trend as those in the former *P*. *dictyopus* species complex, having macromorphological similarities, but being genetically distant. The true phylogenetic relationships withing the “leprieurii” clade require a detailed study among specimens from different regions, especially new collections from the type location. For now, we have identified a new variety that differs from the type specimen on the presence of cystidioles and distinctive hyphae in the trama.

The “guianensis” clade was strongly supported by Maximum Likelihood and Bayesian inferences scores. Unfortunately, it was not possible to use the type specimen in this study. However, reference sequences were used to differentiate our samples. Sequences from Argentina and Venezuela remained as *P*. *guianensis*. Two other sequences from Ecuador (ECU 1 and ECU 2) and the Costa Rica sample seem to be different from *P*. *guianensis* Mont. and *P*. *taromenane* species novo. The genetic difference between our sequences and the reference sequences from Argentina and Venezuela are significant, as they share only 75% sequence identity. Sequences ECU1 and ECU2 and from Costa Rica are closely related to the newly proposed species, *P*. *taromenane* from Yasuní, however, they are still significantly different from *P*. *taromenane*. There are macro and micromorphological similarities between *P*. *taromenane* and *P*. *guianensis*, including stipe position, stipe surface, pilei shape, size of pores, hyphal system, and basidiospores narrowly ellipsoid to cylindrical. However, *P*. *guianensis* is known to be common in tropical regions of Asia, our phylogenetic analysis confirms that these are two distinct taxa.

Taxa belonging to *Picipes* Zmitr. et Kovalenko were included to help reveal the phylogenetic relationships among the taxa sampled in Yasuní and allied genera within the “Melanopus group”. The “picipes” clade represented in this study includes six distinct species from China.

The “neodictyopus” clade includes sequences from Belize, Brazil, and Ecuador with five distinct taxa in the clade. *Neodictyopus* Palacio, Robledo, Reck & Drechsler-Santos, was proposed by Palacio et al. (2017), based on phylogenetic analysis and morphological descriptions of freshly collected and herbarium samples previously identified as *P*. *dictyopus*. Five of the eight *Neodictyopus* samples used in our analysis come from Brazil, two from Yasuní National Park in Ecuador, one from Cuyabeno-Ecuador, and one sample from Belize [45]. *Neodictyopus sylvaticus* species novo shares general morphological features with *N*. *atlanticae*, the type species for the genus. These features include the position of the stipitate, pileus shape and color, hyphal system, and shape of basidiospore. Although, these characteristics can be shared with other taxa within the genus and *Picipes* Zmitr. et Kovalenko (2016). Genetically, *N*. *dictyopus* is the closest taxon with 80% sequence identity, being highly distinctive along the ITS region with only 60% sequence identity. In addition, relevant macro and micromorphological differences were found between *N*. *dictyopus* and *N*. *sylvaticus*, including smaller basidiomata, smaller pores, stipe shape, the presence of ventricose tramal setal, and generative hyphae highly branched, slightly cylindrical along the main stem which are unique characteristics of *N*. *sylvaticus*. We believe these morphological differences and the phylogenetic results support the proposal of this new species. Additionally, in the “neodictyopus” clade, EC-97 herbarium sample previously identified as *P*. *leprieurii* by Laessoe (1995), is now identified as a taxon within the *Neodictyopus* genus. Pilear shape, color and texture; stipe position; and spore characteristics are macro and micromorphological features shared within several taxa in the “Melanopus group”. Since most members have broad morphological plasticity, such characteristics might influence the misidentification of some taxa. As Palacio et al. (2017) pointed out, the color of the pilear surface could be a broad and obvious characteristic to distinguish taxa between the “leprieurii” and “neodictyopus” groups. In the “leprieurii” group, the pilear surface can have orange-ochraceous hues, while the “neodictyopus” group has darker colors of reddish-brown hues. Other microscopical features such as shape and size of spores are broadly shared in the Polyporaceae family, therefore the hyphal system and other structures found in the hymenophore should be considered while identifying a specific taxon in addition to spore descriptions. The “neodictyopus” clades presented by Palacio et al., (2017) and our study include only taxa from the neotropics, and supports the information presented by Palacio et al., (2017) that the genus is neotropical. In our study, the “neodictyopus” clade is closer to “picipes”.

The “atroporus” clade presented in our study includes sequences from Brazil and Ecuador with strong Maximum likelihood bootstrap and Bayesian inference scores. *Atroporus* was proposed by Ryvarden (1973) with *Polyporus diabolicus* Berk. as the species type. It was recently amended by Palacio et al. (2017), using the descriptions of the basidiospores and strongly dextrinoid skeletal-binding hyphae in the trama with a distinctive thick-walled stalk with a pointed apex. In this clade, *A*. *yasuniensis* and *A*. *tagaeri* were two new species described in this study. *A*. *yasuniensis* was closely related to *A*. *rufoatratus*, but with clear genetic and morphological differences. Genetically, *A*. *yasuniensis* and *A*. *rufoatratus* had only 88% sequence identity. Morphologically, *A*. *yasuniensis* had a gregarious habit, big basidiomata, smaller pores, and developed of rhizomorphs compared to the solitary, small basidiome with relatively larger pores and absence of rhizomorphs of *A*. *rufoatratus*. Microscopically, *A*. *rufoatratus* had subulate cystidioles and *A*. *yasuniensis* had ventricose cystidioles.

The second newly described species, *A*. *tagaeri* was genetically different from *A*. *diabolicus*, *A*. *rufoatratus*, and *A*. *yasuniensis* with 83%, 80%, and 84% of sequence identity respectively. Morphologically, *A*. *tagaeri* shared macromorphological features with other taxa in the clade, including centrally stipitate basidiomata with a circular, slightly depressed pileus and the characteristic black cuticle bearing the stipe. Basidiomata of *A*. *tagaeri* were small to medium and pores were smaller and similar to *A*. *yasuniensis*. A remarkable difference between *A*. *tagaeri* and other taxa in the “atroporus" clade was that in *A*. *tagaeri*, the characteristic thick-walled stalk with pointed apex was not found. Considering this is an important characteristic for the description of the genus, it is necessary to carry out additional collections to verify this feature, which was also reported to be present in taxa outside *Atroporus* and has taxonomic value to differentiate those groups. Plectenchymatous thickening of hyphae were rare, but found in *A*. *tagaeri*. These segments of hyphal modifications appeared to be common on *N*. *sylvaticus* and *P*. *taromenane*, and its presence should be used for micromorphological descriptions, but not considered to have taxonomic value. These hyphal modifications have been previously described to be present in *Polyporus* s. str., *Phellinus* and *Daedaleopsis* [24] as well as on several species of lichens, ectomycorrhizal, and other taxa in the Agaricomycetes fungi [46–54]. These hyphal modifications seem to be associated with interlocking hyphae involved in the development of specific tissue accomplishing a specific function. In our results we observed that plectenchymatous thickenings can be common or rare among taxa and can be present along the skeletal-binding hyphae in trama, context, stipe and rhizomorphs. There is still not a clear function of this tissue in the taxa we described, however, it is important to recognize it for future comparisons.

Finally, sequences (ITS, LSU and EF1-α) corresponding to two samples of *P*. *tuberaster* from China were included, as *P*. *tuberaster* is the type species for the *Polyporus* genus, however, the type sequences were not use in this study. This last clade is apparently not related to any of the clades formed in our phylogenetic analysis. The use of genetic data accompanying morphological descriptions has been key to unveil the true taxonomy of several members in *Polyporus* s. l. We believe that more genera will be proposed as additional collections and analyses are carried out in the Amazonian regions of South America.

To accomplish the job of unveiling the true phylogenetic relationships in the “Melanopus group” and other artificial groups within the Polyporaceae family, researchers need to select molecular markers that match reference sequences, generate more sequences of taxa from neotropical, pantropical, and temperate regions and compare them with the taxa of interest.

## Conclusions

We described four new species and one new variety of ecologically important fungi from the Amazon rainforest of Ecuador. Three of these fungi produce aerial rhizomorphs that provide these species with strategies to colonize new substrates and survive in environmentally challenging ecosystems that seasonally exhibit flooding and temporarily drought conditions.

*Polyporus leprieurii* var. *yasuniensis* and taxa within the “leprieurii” subclade are prolific species in the forest at Yasuní National Park where they produce rhizomorphs, often accompanied by basidiomata. While, *Atroporus yasuniensis* has been found producing abundant rhizomorphs and basidiomata growing on the same substrate, this species seems to be rare with only one collection site among all the sampled quadrants and transects we studied. *Polyporus taromenane* is also rare with only three collection sites, however the amount of rhizomorphs it produces is not as abundant as the other species.

Rhizomorphs have not been typically considered to be of taxonomical value. However, in this study we observed that rhizomorphs exhibit morphological characteristics similar to the stipes of basidiomata for each species. This feature helps to differentiate the taxa and can be important for identification when rhizomorphs are found but basidiomata are not present. *Polyporus taromenane* appears to have the most distinctive rhizomorphs and stipe, which has hirsute hairs giving it a velvety appearance. Also, basidiome production seems to be rare in the rainforest and the presence of its rhizomorphs appears to be sufficient to identify this taxon when found.

Macrocromorphological features regarding pileus and stipe shape, texture, color and position are useful to identify taxa within artificial groups and/or fungal families. Although, these features are broadly shared between the groups, some of them may be used to separate some genera, like the color of pileus between *P*. *leprieurii* and *Neodyctiopus*. However, these characteristics are not useful when identifying species complexes. Micromorphological structures should be also carefully considered while identifying and proposing new taxa. For example, spore shape, size, and texture are commonly shared in the examined taxa and in many members within the Polyporaceae family. Other characteristics regarding hyphal types need further revision among more representatives of a specific taxon, as some characteristics like the skeletal-binding hyphae with a distinctive “sharply pointed apex” used to propose *Atroporus* was also found in *P*. *leprieurii* var. *yasuniensis*.

Phylogenetic analyses have become an important tool in taxonomic studies, and we believe this should be used along with morphological descriptions. In this and similar studies, the lack of reference sequences available shows the ongoing need to collect and access genetic material of fungi to create better databases that allow us to understand the true diversity and distribution of these species, especially in highly diverse ecosystems where there is large fungal diversity. Finding enough collections to carry out phylogenetic and taxonomic studies can be challenging when species are rare and inhabit endangered ecosystems. Collaborations among Fungarium and mycological institutions, and researchers are key to access relevant reference sequences and taxa in aims to unveil the true fungal diversity of poorly studied ecosystems and regions such as the Amazon Rainforest of Ecuador.
